# Sympathetic nociceptive afferent signaling drives the chronic structural and functional autonomic remodeling after myocardial infarction

**DOI:** 10.21203/rs.3.rs-6247307/v1

**Published:** 2025-03-31

**Authors:** Marmar Vaseghi, Valerie van Weperen, Jonathan Hoang, Neil Jani, Karim Atmani, Christopher Chan, Kuan Cao, Shail Avathi, Zulfiqar Lokhandwala, Maryam Emamimeybodi

## Abstract

After myocardial infarction (MI), pathological autonomic remodeling, including vagal dysfunction and sympathoexcitation, occurs and predisposes to ventricular arrhythmias (VT/VF). The underlying factors that drive this remodeling, including the observed neuroinflammation and glial activation, remain unknown. We hypothesized that sympathetic nociceptive afferents underlie this remodeling post-MI. Epidural resiniferatoxin (RTX, to ablate sympathetic cardiac afferent neurons) vs. saline was administered in pigs prior to MI and autonomic and electrophysiological effects assessed four to six weeks post-infarction. Acute effects of afferent ablation after chronic MI were also assessed in a separate group of animals. Baroreflex sensitivity and vagal tone, as measured by parasympathetic neuronal activity and cardiac nociceptive responses, were improved in infarcted animals which received epidural RTX prior to MI. These animals also demonstrated reduced spinal cord inflammation and glial activation, downregulation of circulating stress and inflammatory pathways, and stabilization of electrophysiological parameters, with reduced VT/VF-inducibility. Epidural RTX after chronic MI also acutely restored vagal function and decreased VT/VF. These data suggest that cardiac spinal nociceptive afferents directly contribute to VT/VF susceptibility and MI-induced autonomic remodeling, including oxidative stress, inflammation, glial activation, and reduced vagal function, providing novel insights into the causal role of these afferents in driving sympathovagal imbalance after MI.

## INTRODUCTION

Sympathoexcitation and vagal dysfunction, which are known to occur after chronic myocardial infarction (MI), predispose to ventricular arrhythmias (VT/VF), sudden cardiac death (SCD), and result in progression of heart failure[1,2]. Heart failure and MI have been reported to be associated with significant structural and functional pathological autonomic remodeling, including glial activation and inflammation in the peripheral autonomic ganglia[3,4], which are thought to play a role in the sympathovagal imbalance post-MI and contribute to electrophysiological instability and VT/VF. In addition, increased cardiac spinal afferent neurotransmission post-MI has been reported to reflexively amplify sympathetic efferent outflow[5–9], resulting in triggered activity and compounding the exacerbation of ventricular electrophysiological heterogeneities[10–13], leading to life threatening ventricular arrhythmias. Given the multitude of chronic pathological and functional changes noted in the autonomic nervous system after MI and with cardiac injury[1,3,14–16], the factors which initiate this remodeling and underlie the subsequent sympathovagal imbalance (rather than simply associated with the pathology) are unclear. In this study, we hypothesized that cardiac *spinal* (sympathetic) nociceptive afferent signaling underlies and drives the subsequent pathological autonomic remodeling and sympathovagal imbalance after MI, including peripheral autonomic glial activation and inflammatory changes, vagal dysfunction, and systemic oxidative stress, resulting in greater propensity for VT/VF. To evaluate this hypothesis directly, we administered epidural resiniferatoxin (RTX) prior to MI to selectively ablate sympathetic afferents. Four to six weeks after chronic MI, we evaluated the structural/neurochemical changes, functional autonomic responses, and overall electrophysiological stability using a porcine chronic infarct model. Lastly, we evaluated whether spinal sympathetic afferent ablation would be sufficient to acutely improve autonomic balance and reduce VT/VF in chronically infarcted pigs.

## RESULTS

To determine if spinal nociceptive sympathetic afferent signaling drives the pathological structural and functional autonomic remodeling after MI, sympathetic cardiac nociceptive afferents were selectively ablated by administration of thoracic epidural RTX. RTX is an agonist of the transient receptor potential V1, which is expressed by nociceptive afferent neurons, resulting in calcium overload, neuronal cytotoxicity, and cell death[17]. RTX was administered at the epidural C7-T1 level, as the majority of cardiac sensory innervation has been shown to pass through the C7 and upper thoracic dorsal root ganglia (DRG) and spinal horns[18]. To determine safety and efficacy, we first administered epidural RTX in 3 healthy control animals. These experiments demonstrated an initial sympathoexcitatory response after thoracic epidural injection of RTX, with increases in heart rate (HR) and left ventricular systolic pressure (LVSP), which stabilized at approximately 2 hours (Supplemental Figure 1). Therefore, for subsequent animals receiving epidural RTX (cRTX, $n$=11) as well as those undergoing sham injections prior to MI (i.e., epidural catheter placed with saline injection instead of RTX, referred to as sham going forward, $n$=13), infarcts were created 2-3 hours after epidural RTX or saline administration (Figure 1).

Hemodynamic responses to RTX in the epidural cRTX animals prior to MI were similar to our observations in healthy control animals (Supplemental Figure 2). MI-related mortality was not significantly different between sham- and epidural cRTX-treated animals (Supplemental Figure 3). However, significantly more RTX-treated animals developed VT/VF at the time of MI creation, compared to sham animals (Supplemental Figure 3), possibly due to the residual sympathoexcitatory effects associated with epidural RTX administration.

Hemodynamic and autonomic responses, neural activity, and electrophysiological stability were assessed four to six weeks later to evaluate the effects of either epidural sham or cRTX treatment on functional chronic autonomic remodeling. Lastly, we evaluated spinal sensory histopathology and plasma proteomes to further assess the effects of cRTX on molecular markers associated with chronic cardio-autonomic remodeling.

*Confirmation of Sympathetic Afferent Nociceptive Neuronal Ablation*

Spinal nociceptive neurons express and release calcitonin gene-related peptide (CGRP)[19,20]. Therefore, CGRP expression was assessed four to six weeks after MI to ensure nociceptive fiber and neuronal ablation in the C7-T1 dorsal horns of the spinal cord and DRG[21] of sham ($n$=5) and cRTX ($n$=7) animals (Figure 2A-D). In cRTX animals, the percentage of CGRP-immunoreactive neurons in the C7-T1 dorsal root ganglia was significantly decreased (14±2% *vs* 22±3%, respectively, $p$<0.05; Figure 2B), and CGRP-immunoreactivity in the dorsal horn was significantly reduced compared to sham animals (1.2±0.3%

*vs* 5.3±0.6%, respectively, $p<0.05$; Figure 2D), confirming successful chemoablation of nociceptive afferent fibers and neurons in cRTX animals.

*Effects of Chronic Spinal Nociceptive Afferent Ablation Prior to MI on Hemodynamics and Autonomic Responses After Chronic MI*

Hemodynamic parameters including HR, LVSP, inotropy ($dP/dt_{max}$), and lusitropy ($dP/dt_{min}$) were assessed in cRTX ($n$=11) and sham ($n$=13) infarcted animals. Baseline hemodynamic parameters of the animals that received epidural RTX were similar to infarcted sham animals (Figure 3A-D), suggesting no differences in resting state hemodynamics.

We then assessed vagal baroreflex sensitivity (BRS), a marker of parasympathetic function[22,23], by evaluating responses to infusion of phenylephrine. Vagal BRS was significantly increased in cRTX ($n$=11) compared to sham animals ($n$=13; 3.7±1.1 *vs* 0.7±0.2 mmHg/ms, respectively, $p<0.05$; Figure 3E-F). These data suggest that spinal nociceptive afferents play a key role in inducing the vagal baroreflex dysfunction that is observed after MI[22,24].

To further evaluate if sympathetic spinal nociceptive afferents underlie the efferent vagal dysfunction post-MI, the activity of postganglionic parasympathetic neurons in the ventral interventricular ganglionated plexus (VIVGP), the cardiac ganglion that provides significant parasympathetic innervation to the ventricles[25-27], was recorded from epidural cRTX animals ($n$=6) and sham animals ($n$=5; Figure 3G). Post-ganglionic parasympathetic neurons in the VIVGP were defined as neurons that showed a statistically significant alteration in their vagal activity in response to low level cervical vagus nerve stimulation (VNS)[28,29]. Low level cervical VNS allows for assessment of neuronal activity without eliciting hemodynamic changes that can lead to reflex autonomic activation[28-30], thereby enabling the classification and evaluation of neurons based on their vagal input. Spinal nociceptive afferent blockade prior to MI did not affect overall bulk firing rates of the neurons in the VIVGP (Figure 3H) or the firing rate of VNS non-responsive neurons (Figure 3I). However, the baseline firing rates of VNS-responsive neurons (i.e., post-ganglionic parasympathetic neurons) was significantly higher in chronically infarcted animals treated with epidural RTX prior to MI compared to sham-treated animals (Figure 3J). This, along with the BRS data, suggested that interruption of spinal sympathetic afferents prior to MI prevents vagal dysfunction post-MI, indicating that nociceptive afferents are at least in part, directly responsible for the reduction in vagal tone observed after MI.

To further test reflex-driven autonomic responses, bradykinin and capsaicin were applied to the ventricular epicardium to activate cardiac nociceptive afferent nerve endings. While epicardial bradykinin elicited a sympathetic response in sham animals ($n$=10), epidural cRTX animals ($n$=11*)* exhibited a predominantly vagal response characterized instead by decreases in HR and LVSP ($p<0.05$ for all parameters, Figure 4A-B). Notably, repolarization time prolonged and dispersion of repolarization (DoR) was significantly decreased in response to bradykinin in animals that had received epidural RTX *vs* sham prior to MI ($p<0.05$; Figure 4C-D). It is known that sympathetic stimulation can increase DoR in healthy hearts[31]. However, following MI, denervation and heterogeneous nerve sprouting at scar and border zone regions occurs[10,32,33]. With sympathetic activation, therefore, electrical heterogeneity is further amplified, resulting in greater DoR, which acts in tandem with structural myocardial changes to produce the electrical substrate for VT/VF[34-37].

Like bradykinin, capsaicin increased LVSP, LV inotropy, and DoR in sham animals, consistent with a predominantly sympathetic and pro-arrhythmic response. However, in epidural cRTX animals, these effects were reversed, and a more vagal response with reduced DoR was observed, potentially consistent with reduced arrhythmia susceptibility (Figure 4E-H). Therefore, these experiments further confirmed that cardiac spinal afferent ablation prevents the development of exaggerated adrenergic reflexes in response to nociceptive activation and improves vagal reflexes post-MI.

*Role of Spinal Afferents in Systemic Oxidative Stress and Inflammatory Response*

Another hallmark of cardiovascular disease is increased systemic inflammatory responses and oxidative stress[16,38,39]. Thus, to assess if this inflammatory response is driven, at least in part, by sympathetic nociceptive afferents, the plasma proteome was examined by mass spectrometry in epidural cRTX ($n$=10) and sham treated ($n$=9) chronically infarcted animals (Figure 5A). Differentially expressed proteins in epidural cRTX *vs* sham animals were found to be primarily associated with pathways encompassing the immune response, oxidative stress, and cell cycle regulation (Figure 5B; Supplementary File 1). Notably, PSMD8, ZAP70 and VAV1, key proteins in the regulation of immunity[40-43], were all downregulated in animals that received epidural RTX prior to MI. Moreover, pathways associated with immune activation and oxidative stress were diminished in animals treated with epidural cRTX (Figure 5C; Supplementary File 2). Conversely, epidural cRTX animals exhibited elevated levels of proteins in the ERK1 and ERK2 cascade, pathways which have been described to be fundamental for cardiomyocyte homeostasis[44].

To further assess the effects of spinal afferent ablation on peripheral sympathetic inflammation and glial activation four to six weeks after MI, the overall immunoreactivity for GFAP, IBA1, and CD3 were assessed in the dorsal horn of the spinal cord of animals treated with epidural RTX ($n$=7) vs sham ($n$=5) prior to MI. In accordance with the changes observed by plasma proteomics, which suggested a decrease in inflammatory pathways in epidural cRTX animals, we also observed decreases in GFAP, IBA1, and CD3 immunoreactivity (Figure 6A-E), animals that had undergone nociceptive afferent depletion prior to MI. Similarly, glial activation was significantly decreased in the T1 DRG of epidural cRTX ($n$=5) *vs* sham ($n$=5) animals (Supplemental Figure 4).

*Effect of Spinal Nociceptive Afferent Ablation Prior to MI on Electrophysiological Parameters and Ventricular Arrhythmias*

Detailed electrophysiological measurements were evaluated in epidural cRTX ($n$=11) *vs* sham animals ($n$=13) to determine if epidural RTX prior to MI had affected the chronic global and/or regional heterogeneity in action potential duration reported after chronic MI. For regional analyses, ARIs were compared between scar, border zone and viable regions, as determined by epicardial voltage mapping[30,45]. Baseline (resting) global ventricular ARIs and regional ARIs were not significantly different between infarcted epidural cRTX *vs* sham animals (Figure 7A-C). Moreover, basal ventricular global DoR was not significantly different between epidural cRTX *vs* sham animals (Figure 7D). However, regional analyses demonstrated that the border zone dispersion was significantly greater in sham *vs* epidural cRTX animals (984±234 *vs* 351±154 $ms^2$, respectively, $p$<0.05; Figure 7E). While atrial effective refractory period (ERP) was not significantly different (Figure 7F), ventricular endocardial ERP (measured at the right ventricular apex) was significantly longer in infarcted animals treated with epidural cRTX *vs* sham (283±5 *vs* 267±5 msec, respectively, $p$<0.05, Figure 7G-H), suggesting increased ventricular refractoriness. Finally, DoR of the prematurely paced beat (S2), which is known to activate sympathetic mechanoreceptors and reflexively increase sympathetic outflow resulting in ventricular arrhythmias[46,47], was significantly less in epidural cRTX animals (epidural cRTX: 817±68 *vs* sham: 1163±90 $msec^2$, $p$<0.05, Figure 7I). Importantly, VT/VF was less inducible in RTX-treated animals, with 62% of sham treated *vs* 18% of epidural cRTX infarcted animals being inducible ($p$<0.05; Figure 7J-L).

*Acute Hemodynamic and Autonomic Responses to RTX After MI*

Given that longitudinal assessments of epidural RTX suggested an important role for spinal sympathetic afferent neurotransmission on autonomic remodeling post-MI, we next sought to assess whether acute administration of epidural RTX could still mitigate the already established autonomic deficits. To test this hypothesis, in a separate group of chronically infarcted animals (four to six weeks post-MI), we assessed hemodynamic and autonomic function in response to acutely administered epidural RTX (aRTX, $n$=15). Acute hemodynamic profiles were continuously assessed *pre*- and *post*-epidural RTX administration. Hemodynamic parameters initially demonstrated sympathetic responses with peak increases in heart rate (84±4 to 87±4 beats/min, $p$=0.001), LVSP (118±5 to 131±7 mmHg, $p$<0.01), and inotropy (1393±67 to 1484±79 mmHg/s, $p$<0.01) within one hour, Figure 8. Four hours after epidural RTX administration, HR remained significantly elevated, while LV inotropy and LVSP returned to near baseline (Figure 8A-E).

While hemodynamic parameters peaked within the first hour after RTX, no effect on BRS was observed at one hour (from 1.8±0.2 pre-RTX to 1.7±0.4 mmHg/ms 1-hour post-RTX, $p$>0.05; $n$=10, Figure 8F-G). However, at four hours after RTX administration, BRS had significantly increased to 3.9±0.7 mmHg/ms ($p$<0.05), suggestive of augmented vagal function.

*Effects of Acute Epidural RTX on Ventricular Refractoriness and Arrhythmia Susceptibility Post-MI*

While previous studies have demonstrated the clinical anti-arrhythmic efficacy of thoracic epidural anesthesia using lidocaine or bupivacaine[48,49], the differential contributions of efferent *vs* afferent blockade to ventricular arrhythmias remained unclear. Furthermore, whether spinal afferent blockade alone could be anti-arrhythmic is unknown. Hence, the acute effects of spinal sympathetic afferent nociceptive ablation on cardiac electrical stability were evaluated before and after epidural RTX

administration in chronically infarcted animals. Four hours after RTX administration, significant shortening of global ventricular ARIs was observed (374±16 pre-RTX to 345±14 msec post-RTX; $p<0.05$, Figure 8H). However, ARI corrected for heart rate (ARIc) was unchanged, suggesting a HR-driven effect, Figure 8I. While atrial ERP remained unchanged ($p>0.05$; Figure 8J), right ventricular endocardial ERP significantly increased from 246±5 pre-RTX to 258±4 msec post-RTX ($p<0.05$), Figure 8K. Moreover, while epidural RTX had no effect on resting/sinus rhythm DoR, it significantly decreased DoR of the ventricular extra-stimulus paced beat (S2), which simulates an early PVC and is typically used to induce VT/VF (1639±130 pre-RTX *vs* 1389±112 $msec^2$ post-RTX, $p<0.05$, Figure 8L-M). Paced ventricular beats and PVCs are known to increase reflex sympathetic efferent responses by activating afferent fibers, increasing DoR[46,47]. No animals developed spontaneous ventricular arrhythmias upon administration of epidural RTX, despite the initial sympatho-excitation and prior infarct. However, only 3 of 12 animals treated with acute epidural RTX were inducible for VT/VF at 4 hours post-RTX administration, which was significantly fewer than the infarcted animals treated with sham RTX prior to MI (25%. *vs* 62%, respectively; $p<0.05$; Figure 8N).

## DISCUSSION

*Major Findings*

The present study demonstrates that cardiac spinal nociceptive afferent signaling is a critical factor in initiating and driving the chronic pathological autonomic remodeling after MI, including chronic vagal dysfunction, neuroinflammation and glial activation, upregulation of systemic inflammatory and oxidative stress pathways, and electrophysiological instability leading to ventricular arrhythmias. These results shed light on a potentially important mechanism underlying the multitude of chronic pathological autonomic remodeling processes that have been described after MI[1-3,14,16,50-52]. Furthermore, acute ablation of these fibers, even after chronic MI, established a more electrically stable substrate and enhanced vagal tone.

*Autonomic Remodeling Post-MI*

MI is associated with a plethora of processes collectively described as pathological autonomic neural remodeling and dysfunction, which culminate in vagal withdrawal and sympathoexcitation[1-3,14,16,50-52].

At the time of cardiac injury, increased sympathetic afferent signaling results in sympathetic efferent activation, in an attempt to restore cardiac output. However, persistent chronic cardiac sympathetic afferent signaling after MI has been reported to maintain the increases in sympathetic outflow to the heart that is thought to be chronically detrimental, resulting in ventricular dysfunction[5,53-55], and ablation of these afferents was reported to improve ventricular function in heart failure rats[5,9]. Additionally, MI is also associated with vagal dysfunction and decreased basal activity of post-ganglionic parasympathetic neurons in the cardiac plexi[30], changes that are thought to result from concomitant reductions in central vagal drive and increases in sympathetic tone[56,57]. Prior studies in heart failure rats and in a porcine infarct model using thoracic epidural anesthesia suggested that spinal afferents decrease baroreflex sensitivity[5,29]. Hence, in this study, we aimed to evaluate if spinal nociceptive afferent signaling *underlies and drives* the exaggerated sympathetic responses and reduced vagal tone and structural autonomic remodeling reported after MI in a clinically relevant large animal model. Responses to epicardial application of capsaicin, a nociceptive transient vanilloid receptor agonist[58], and bradykinin, an endogenous ligand for kinin B2 receptors released during myocardial ischemia[59], were compared between sham and epidural cRTX animals. Sham animals demonstrated an overt sympathoexcitatory response, whereas in epidural cRTX, a predominantly vagal response was observed, suggesting improvements in vagal tone. These findings are in line with a previous study in healthy rats which reported that epicardial application of RTX mitigated capsaicin- and bradykinin-induced increases in renal sympathetic nerve activity[58]. However, while RTX is chemically selective, its epicardial application is anatomically non-selective, as it can also ablate cardiac vagal TRPV1 afferents, which are thought to exert cardioprotective effects[60,61]. The more pronounced vagal responses observed after capsaicin and bradykinin application in this study, might, therefore, reflect the intact vagal reflexes that were elicited. These results suggest that the absence of chronic nociceptive signaling mitigates sympathetic activation and improves vagal function after MI, reducing the electrophysiological heterogeneity that predisposes to VT/VF.

Reduced vagal function, as assessed by BRS, has been demonstrated to be an independent predictor of SCD and VT/VF in patients with MI and heart failure[22,24]. In this study, animals with nociceptive spinal afferent ablation prior to MI demonstrated improved vagal BRS four to six weeks after infarction. Moreover, in these animals, the activity of VIVGP neurons that receive central vagal inputs was also significantly higher than in sham animals, demonstrating a greater central efferent vagal drive to these intrinsic cardiac parasympathetic neurons. Taken together, these data suggest that spinal nociceptive afferents play a critical role in the subsequent vagal dysfunction after MI.

*Molecular Remodeling of Autonomic Ganglia and Inflammatory Responses*

MI has also been reported to be associated with molecular remodeling and inflammation of autonomic ganglia, including glial activation and oxidative stress[16,39,62-64]. However, anti-inflammatory therapies such as TNF-a, IL-1β, and IL-6 inhibitors have been met with mixed results in patients with MI[65-67]. In this study we hypothesized that cardiac spinal afferent signaling precedes and drives this adverse inflammatory remodeling and oxidative stress and evaluated if ablation of these afferents prior to MI could mitigate this systemic response. Proteomic analyses of plasma from epidural cRTX and sham animals and histological assessment of spinal cord dorsal horns and DRGs demonstrated that the markers of inflammation and stress were lower in RTX treated compared to infarcted animals that received saline. Interestingly, we found that SMAD signaling – a pathway implicated (especially through SMAD3) in infarct healing[68-70] – was also increased in epidural cRTX animals compared to sham. Inhibition of SMAD3 has been reported to lead to perturbation of fibroblast alignment and disorganization of scars[68,69], which might in turn predispose to further electrical heterogeneity and ventricular arrhythmias.

Within the central nervous system, microglia migrate towards the site of injury or active inflammation[71] while T lymphocytes cross the blood brain barrier to reach these sites[72]. In the dorsal horn, a lower immunoreactivity for microglial and T lymphocyte markers, IBA1 and CD3, respectively, was observed in epidural cRTX animals. Moreover, glial activation, which has been reported in the stellate and DRGs of both small and large animal models of chronic MI and in patients with heart failure and VT/VF[3,73], was significantly attenuated in the spinal cord and DRG of animals treated with RTX. Prior studies have shown that spinal cord astrocytes become reactive in response to cardiac ischemia[63,74], and their inhibition could potentially reduce susceptibility to arrhythmias[63]. In this context, our results suggest that spinal nociceptive afferent signaling may underly and drive, at least in part, the associated systemic inflammatory and stress responses as well as molecular adaptations, such as glial activation, in the spinal cord and dorsal root ganglia, after MI.

*MI-induced Electrophysiological Changes that Predispose to VA*

Cardiac autonomic dysfunction has been shown to play an established role in the genesis and maintenance of ventricular arrhythmias after MI[1,75,76]. Acute MI causes myocardial injury and axonal denervation[77], followed by localized nerve sprouting, especially in border zone regions [52]. In the setting of sympathoexcitation, these structural adaptations lead to significant electrical heterogeneity in action potential duration and refractoriness, especially in border zone regions, serving as the substrate for VT/VF[13,78,79]. In addition, MI also induces electrical remodeling of myocytes, including an increase in calcium current densities and a decrease in potassium current densities[80]. Sympathetic activation in both healthy and diseased hearts has also been shown to cause early and late depolarizations[34,76,81], serving as the trigger for VT/VF.

We hypothesized that interruption of spinal/sympathetic nociceptive afferents, by maintaining sympathovagal balance after MI, would reduce VT/VF susceptibly. In our study, more RTX-treated animals developed VT/VF during MI creation, possibly due to the initial, heightened sympatho-excitatory state caused by RTX administration prior to complete ablation of these neurons. However, mortality rates were not different (Supplemental Figure 3). Notably, in cRTX treated animals, border zone regions demonstrated a reduction in DoR, and therefore, electrical heterogeneity, consistent with anti-arrhythmic effect. In fact, following chronic MI, the reductions in VT/VF-inducibility in epidural RTX treated animals were surprisingly comparable to the anti-arrhythmic benefits reported with thoracic epidural anesthesia using bupivacaine[82] and cardiac sympathetic denervation in patients with refractory VT/VF and electrical storm[83,84], interventions that interrupt both sympathetic efferent and afferent sympathetic fibers. In addition, several electrophysiological and hemodynamic differences between epidural cRTX *vs* sham animals were only observed in the setting of interventions known to activate sympathetic afferents and cause sympathoexcitation, such as ventricular pacing and application of capsaicin/bradykinin[47,58,85,86].

A prior study had examined the electrophysiological effects of ablation of cardiac TRPV1 neurons via application of RTX on the dorsal root ganglia and reported reduced VT/VF during acute ischemia[87]. In our study, the observed anti-arrhythmic effects after chronic MI were likely to be, at least in part, due to the 1) decrease in systemic inflammatory and stress responses and spinal glial and inflammatory changes, 2) improvements in central vagal drive, with resulting reduction in electrophysiological heterogeneity of border zone regions that are known to trigger and serve as the substrate for VT/VF. Modest improvements in vagal tone post-MI have been previously reported to reduce ventricular arrhythmias in large animal models[88,89].

*Acute Suppression of the Pro-Arrhythmic Phenotype After Chronic MI*

Given that the data from the epidural cRTX animals suggested that cardiac sympathetic afferents play a fundamental role in inducing post-MI autonomic remodeling, including vagal dysfunction, we sought to determine whether delayed intervention with RTX four to six weeks after MI could, at least acutely, restore vagal function and reduce arrhythmia susceptibility. Our data demonstrated that acute RTX in infarcted animals improved vagal BRS, increased ventricular refractory period, mitigated pacing-induced dispersion of repolarization, and decreased VT/VF inducibility at four hours after administration. Hence, cardiac sympathetic afferents are likely to not only precede and induce post-MI structural remodeling processes, but also appear to maintain the pathological vagal dysfunction after MI.

*Clinical Implications*

RTX is a potent, selective, transient receptor potential vanilloid 1 (TRPV1) agonist, a receptor that is highly expressed by nociceptive neurons[58] that results in cell death due to cytotoxicity from calcium activation[17]. RTX is currently being investigated in clinical trials of patients with chronic and refractory pain (NCT00804154; NCT02522611), highlighting its clinical potential.

In addition to showing that nociceptive spinal afferents underlie and induce many aspects of the autonomic remodeling observed in the setting of chronic MI, our study demonstrates the anti-arrhythmic potential of selective cardiac spinal afferent ablation. It is important to note that none of the animals (infarcted or healthy) experienced hemodynamic instability after epidural RTX. However, initial administration of RTX was associated with transient sympathoexcitation that peaked by 1-hour and increased susceptibility to ventricular arrhythmias at the time of acute MI. Hence, if administered during acute MI, other agents, such as anesthetics, may have to be co-administered to mitigate these initial effects. Notably, the significant anti-arrhythmic effects of epidural RTX four to six weeks post-MI highlight the underlying role of cardiac spinal afferents in the occurrence of VT/VF and sympathovagal imbalance. Additionally, our acute epidural RTX studies demonstrate the ability of spinal afferent ablation to improve cardiac autonomic balance in chronically infarcted animals. Future studies are warranted to further determine the extent to which epidural RTX after chronic MI can *reverse* pathological cardiac autonomic remodeling.

***Limitations***

RTX was administered epidurally without *acute* confirmation of ablation of nociceptive afferents. However, sympathoexcitatory responses upon initial administration were observed in all animals, and histological analyses four to six weeks post-MI demonstrated decreased CGRP immunoreactivity, indicative of nociceptive afferent neural ablation.

Given epidural administration at C7-T1 vertebral levels, effects of RTX in this study may represent an underestimate of its potential efficacy, as complete blockade of *all* cardiac spinal afferent nerves was difficult to confirm. However, the blockade herein was sufficient to mitigate VT/VF inducibility and reduce post-MI remodeling.

## CONCLUSIONS

Cardiac spinal/sympathetic nociceptive afferent signaling plays a causal role in the occurrence of the multitude of reported, pathological autonomic changes after chronic MI. In chronically infarcted animals, ablation of spinal cardiac afferents prior to MI prevents MI-induced vagal dysfunction, glial activation and inflammatory changes, and electrophysiological instability. Moreover, acute RTX administration in chronically infarcted animals improved vagal tone and reduced inducibility for ventricular arrhythmias. These results offer important insights on the mechanisms underlying the chronic pathological autonomic remodeling and susceptibility to ventricular arrhythmias observed after MI.

## Methods

*Ethical Approval*

Animal care was performed according to the National Institutes of Health Guide for the Care and Use of Laboratory Animals. All experimental protocols were approved by the UCLA Institutional Animal Care and Use Committee.

In total, 57 male Yorkshire pigs (S&S Farms) were included in the study. Of these, three were healthy animals (Control; *n*=3) initially used to assess hemodynamic effects of acute epidural RTX. Of the other infarcted 54 animals, 42 pigs survived the MI creation and the four-to-six-week post-MI survival period for terminal studies. Nineteen animals were randomized to epidural cRTX, of which 11 survived the acute MI and the four-to-six-week period post-MI, and 18 animals were randomized to sham (underwent thoracic epidural catheter placement without RTX administration), of which 13 survived the acute MI and the four-to-six-week period following MI. A fourth group of 17 animals underwent MI creation, of which 15 survived the acute MI and the four-to-six-week period post-MI and were used for evaluation of the effects of acute RTX in chronically remodeled infarcted animals, Figure 1A. In epidural cRTX and sham animals, terminal experiments were performed four to six weeks after epidural RTX injection and MI creation. Sham animals had an epidural catheter placed and contrast/saline injected without RTX, 2-3 hours prior to MI (Figure 1). In aRTX animals, hemodynamic and electrophysiological parameters were assessed four to six weeks after MI, before and up to four hours after administration of epidural RTX. Not all animals underwent the same interventions at the time of terminal studies due to the prolonged length of experiments and potential for micro- or macro-dislodgment of neural recording electrodes from the VIVGP with cardioversion of VT/VF inducibility or rinsing with saline after epicardial bradykinin/capsaicin application. Hence animal numbers are indicated with each intervention under each section of the results.

*Creation of Myocardial Infarcts*

MI was created percutaneously under fluoroscopic guidance, as previously described.[30] Briefly, animals (40.4±0.7 kg) were sedated with tiletamine-zolazepam (4-8 mg/kg, intramuscular), intubated, and placed under general anesthesia (isoflurane 1-2%, inhaled). A coronary guide wire was introduced *via* the femoral artery into the left anterior descending coronary artery (LAD), and a balloon-tipped angioplasty catheter was advanced over an angioplasty wire past the first diagonal branch of the LAD. Next, the balloon was inflated and 3-4 ml polystyrene microspheres (Polybead, 90 μm, Polysciences, Warrington, PA) were injected through the angioplasty balloon lumen into the distal LAD. The balloon was deflated, and MI confirmed by lack of distal LAD flow on coronary angiography coupled with ST-segment changes (Figure 1B-C). Animals were then survived for four to six weeks.

*High Thoracic Epidural Afferent Denervation*

Pigs were placed in the lateral decubitus position. Using a paramedian approach, a 17-gauge Tuohy needle was inserted at the T5-T6 vertebral level, and the epidural space identified by standard loss-of-resistance under fluoroscopic guidance. Correct placement of the catheter in the epidural space at the C7-T1 position were then confirmed by contrast injection under fluoroscopic guidance. A 19-gauge open-end epidural catheter (Teleflex Inc, Wayne, PA) was then advanced superiorly to the C7-T1 vertebral level (Figure 1F) and contrast again injected to confirm position. RTX (0.6-1.2 μg/kg; 1 mL) or saline with contrast was injected into the epidural space.

*Animal Preparation*

For terminal studies, animals (49.8±0.1 kg) were sedated with tiletamine-zolazepam (4-8 mg/kg, intramuscular) and intubated. General anesthesia was induced with isoflurane (1-2%, inhaled) and transitioned to α-chloralose (50 mg/kg initial bolus, then 20-30 mg/kg/hr infusion) prior to autonomic or electrophysiological testing. Sheaths were placed in bilateral femoral veins and arteries for saline infusion, drug administration, pressure monitoring, and introduction of electrophysiological catheters.

*Ventricular Hemodynamic Measurements*

A 5-Fr Millar pressure-conductance catheter was introduced *via* the femoral artery and placed in the left ventricle (LV) for continuous pressure. Raw signals were digitized by a CED Power1401 and analyzed using Spike2 (Cambridge Electronic Design). A continuous 12 lead ECG was obtained via a CardioLab Recording System (GE Healthcare).

*Ventricular Electrophysiological Measurements*

All animals underwent median sternotomy to expose the heart. A 56-electrode sock, connected to a GE CardioLab system, was placed over the ventricles for continuous local unipolar epicardial electrogram recordings (band pass filtered 0.05-500 Hz, Figure 1D). Activation time (AT) and repolarization time (RT) were measured by customized software (iScaldyn; University of Utah) from these unipolar electrograms as the intervals from onset of ventricular activation to the minimal dV/dt of the depolarization wave-front or maximal dV/dt of the repolarization wave-front, respectively (Figure 1E). Activation recovery interval (ARI), a surrogate for action potential duration,[90] was calculated as the difference between RT and AT, and corrected for differences in HR using the Bazett formula. Global dispersion in RT (DoR) was calculated as the RT variance across all sock electrodes, whereas regional DoR was calculated as the variance across electrodes assigned to regions based on bipolar voltage mapping (below).

Bipolar voltage mapping was performed in epidural cRTX (*n*=11) and sham (*n*=13) using a standard 2-2-2 duodecapolar catheter (Abbot, Minneapolis, MN) to delineate scar, border zone and viable regions. For this purpose, the duodecapolar catheter was advanced between the epicardium and the sock electrode and the bipolar voltage underneath each respective electrode was measured. Using standard voltage criteria regions were defined as either scar (0.05 mV<voltage<0.5 mV), border zone (0.5 mV<voltage<1.5 mV), or viable (voltage>1.5 mV) myocardium[45].

*Evaluation of Cardiac Autonomic Function*

<u>Baroreflex sensitivity</u>

Vagal baroreflex sensitivity was tested in sham animals (epidural with saline/contrast only followed by MI; *n*=13), epidural cRTX animals (epidural with RTX followed by MI, *n*=11), epidural aRTX (MI followed by epidural RTX four to six later; *n*=12) by bolus injection of phenylephrine (3-5 μg/kg, IV) to evoke a 30-40 mmHg increase in systolic pressure. Vagal BRS was measured hourly for four hours in epidural aRTX animals, while it was measured once in control, sham and epidural cRTX animals. The slope of the linear regression describing the beat-to-beat relationship between RR-interval and LV systolic pressure (LVSP) was used to quantify baroreflex sensitivity (BRS)[91].

<u>Cardiac nociceptive stimulation</u>

Epicardial application of bradykinin (1.06 mg/mL) and capsaicin (0.03 mg/mL) in sham (*n*=10) and epidural cRTX (*n*=11) animals was used to characterize autonomic responses to epicardial stimulation of chemosensitive, nociceptive afferents. Chemicals were individually applied (15 mL over 10 seconds) and thoroughly washed off with 500 mL of warm saline 1-minute after start of application. A 15-30 min wait period was allowed for hemodynamic parameters to return to baseline prior to subsequent interventions.

### *Extracellular Neural Recording from the Intrinsic Cardiac Nervous System*

Custom-made 16-channel linear microelectrode arrays (MicroProbes for Life Science; 25-μm-diameter platinum/iridium electrodes, 16 electrodes/probe, 375-μm interelectrode spacing) were used for *in vivo* extracellular neural recordings of the VIVGP (Figure 4), as previously described[29,30,73] in 5 sham and 6 epidural cRTX animals. In short, the probe was gently advanced into the epicardial fat pad and serially connected to a head-stage preamplifier and a 16-channel preamplifier (Model 3600, A-M Systems, Sequim, WA). All signals were continuously recorded and digitized (Cambridge Electronic Design) at a sampling frequency of 20 kHz, and band-pass filtered (0.3 - 3 kHz). Offline processing and analyses of neural signals was performed using Spike2 software (Cambridge Electronic Design), as previously described[29,30,73]. Artifacts (recognized as simultaneous waveforms on all neural recording channels) were removed, and neuronal spikes were identified using a threshold of 2 times signal-to-noise ratio. Spike sorting was performed using principal components, cluster on measurements, and K-means clustering analysis to identify unique neuronal waveforms[28,92]. Efferent postganglionic parasympathetic neurons in the VIVGP were identified based on their responses to left or right-sided VNS. Briefly, bipolar spiral cuff electrodes (LivaNova, PLC) were placed around each cervical vagus, and the VIVGP activation threshold current, defined as the VNS current needed to evoke a 10% decrease in heart rate (20 Hz, 1 ms), was determined. Each cervical vagus was then stimulated separately for 1 minute at 1 Hz (1 ms, 1× VIVGP activation threshold current). At least 20 minutes of recovery time was allowed between the two stimulations. Baseline activity during the 2 minutes before left or right VNS was compared with the 2 minutes at the start of stimulation (1 minute during VNS and 1 minute after) using the Skellam statistical test[93]. Neurons that showed significant changes in firing activity (as compared by the Skellam test) upon left or right VNS were identified as postganglionic parasympathetic neurons, while neurons that did not show significant alterations in their activity were considered to be non-VNS responsive. Basal activity of both VNS-responsive and non-VNS responsive (1 minute) was compared between sham ($n$=5) and epidural cRTX ($n$=6) animals.

#### *Effective Refractory Period Measurements and VT/VF Inducibility*

Atrial and ventricular effective refractory periods (ERP) were measured by extra-stimulus pacing at a drive cycle length (CL) of 450 msec, with S2 decremented by 5 msec, using a pacing catheter placed on the epicardial left atrial appendage and in the right ventricular (RV) apex from the right femoral vein.

VT inducibility was tested in epidural cRTX ($n$=11), sham ($n$=13), epidural aRTX ($n$=12) animals. Inducibility was assessed by programmed stimulation (as is standard in electrophysiology laboratories for testing of inducibility in patients with heart disease)[94] by an 8-beat drive train (at CL of 450 ms) followed by an S2 extra-stimulus, which was decremented by 10 msec down to a CL of 200 msec or ERP, whichever occurred first. If no VT/VF was induced, a CL of 20 msec above ERP was selected for the extra-stimulus to ensure ventricular capture and the next extra-stimulus (up to S4) was added. VT/VF inducibility was defined as the occurrence of sustained VT (>30 seconds) or VF requiring defibrillation. Inducible animals were cardioverted if VT/VF did not terminate after 30 seconds. VT inducibility was tested from RV endocardium and, if non-inducible, also from the LV anterior epicardial border zone region. For acute RTX animals, the same site that induced VT/VF pre-RTX administration was used post-RTX administration to induce VT. Ventricular pacing threshold were checked and maintained pre- *vs* post-RTX administration (Supplemental Figure 5).

*Histopathological Assessment*

C7-T1 spinal cords were collected from epidural cRTX (*n*=5-7) and sham animals (*n*=5), fixed in 4% paraformaldehyde, and embedded in paraffin. Tissue was deparaffinized, rehydrated, and epitopes unmasked at 90 °C in EDTA buffer (Abcam, ab64216). Slides were blocked and incubated overnight at 4 °C with goat anti-calcitonin gene-related peptide (CGRP; 1:1000; Abcam, ab36001), mouse anti-glial fibrillary protein (GFAP; 1:1000; Invitrogen, ASTRO6), rabbit anti-ionized calcium-binding adaptor molecule 1 (Iba1; 1:1000; Biocare Medical, CP 290 A, B), and/or rat anti-cluster of differentiation 3 (CD3; 1:1000; Abcam, ab11089). CGRP was used as a surrogate for TRPV1 expression as it is released by nociceptive sensory/afferent neurons upon TRPV1 activation[95]. Sections were incubated for 2 hours at room temperature with Alexa Fluor 488–donkey anti-goat IgG (1:200; Invitrogen, A-11055), Alexa Fluor 555–donkey anti-mouse IgG (1:400; Invitrogen, A-31570), Alexa Fluor 488–donkey anti-rabbit IgG (1:400; Invitrogen, A21206), and/or CF405S–donkey anti-rat IgG (1:400; Biotium, 20419), respectively and mounted with Antifade Mounting Medium (Vector Laboratories, H-1000-10). Slides were imaged on a Zeiss LSM880 at 20×, and 63× magnifications and processed with Zen 2 software (Zeiss). Fractional area of dorsal horn CGRP immunoreactivity quantified using ImageJ software (NIH). Spinal cord GFAP, IBA1 and CD3 immunoreactivity (quantified as number of immunoreactive cells in the dorsal horn divided by the total dorsal horn area ×100) was assessed and quantified using ImageJ. In addition, glial activation and CGRP immunoreactivity was also evaluated in the C7-T1 dorsal root ganglia using ImageJ.

*Proteomics*

Sample Preparation

After access to the femoral artery was obtained and prior to performing any autonomic or electrophysiological testing, baseline blood was collected in EDTA blood tubes and immediately placed on ice. Samples were centrifuged and plasma was separated and stored in -80 °C. Plasma samples were prepared by the University of California, Los Angeles (UCLA) Proteome Research Center, using the Mag-Net[96]. Digested peptides were separated online using C18 reversed phase chromatography on a ThermoFisher Vanquish Neo UHPLC. MS/MS spectra were collected using a data-independent analysis (DIA) acquisition method on ThermoFisher Orbitrap Astral mass spectrometer[97,98]. Data were analyzed using the DIA-NN algorithm in which peptide and protein identifications were filtered using an estimated false discovery rate of less than 1%[99]. Comparison testing between conditions was performed using Fragpipe-Analyst platform using DIA-NN-generated label-free protein abundances[100].

Data Analysis

Data analysis was performed in R. Raw counts were normalized with random-forest normalization using R. Differentially expressed proteins were identified using a two-sided Student’s t-test. Proteins with a *p-value < 0.05* were considered statistically significant, and those with *|log2(fold change)| > 0.5* were identified as differentially expressed proteins. Pathway analysis was performed against the Gene Ontology (GO) database using Rapid Integration of Term Annotation and Network (RITAN, v3.20)[101]. A two-sided *p*-value < 0.05 was used to determine statistically overrepresented pathways.

*Statistical Analysis*

Data are presented as mean ± SEM. After confirmation of normality, paired two-tailed Student’s *t*-test or Wilcoxon signed rank test was used to compare pre- and post-RTX parameters in epidural aRTX animals, depending on Gaussian distribution. Immunohistochemical data was analyzed using unpaired Student’s t-tests or Mann-Whitney U test (depending on Gaussian distribution). Unpaired analysis of variance (ANOVA) was used for intergroup comparisons (sham *vs* epidural cRTX *vs* Control) and changes in hemodynamic parameters over time were compared using repeated measures ANOVA. Electrophysiological parameters were compared using unpaired Student’s t-tests or Mann-Whitney U test (depending on Gaussian distribution) for epidural cRTX *vs* sham animals or repeated measures ANOVA in case of serial comparisons in acute RTX studies. BRS data was only included for analysis if $R^2$ was greater than 0.8 for the slope of the regression line relating blood pressure to heart rate and compared using the Mann-Whitney U test (for group comparisons) or the Friedman test (for serial comparison in acute RTX experiments). Comparison of VT/VF inducibility was performed using the binomial exact test. *P-value* < 0.05 was considered statistically significant.

## Abbreviations

ARI = activation-recovery interval

AT = activation time

BRS = baroreflex sensitivity

CD3 = cluster of differentiation 3

CGRP = calcitonin gene-related peptide

CL = cycle length

DoR = dispersion of repolarization time

ECG = electrocardiogram

ERP = effective refractory period

GFAP = glial fibrillary acidic protein

HR = heart rate

LAD = left anterior descending

LV = left ventricle

LVSP = left ventricular systolic pressure

MI = myocardial infarction

RT = recovery time

RTX = resiniferatoxin

SCD = sudden cardiac death

TRPV1 = transient receptor potential vanilloid 1

VIVGP = ventral interventricular ganglionated plexus

VNS = vagal nerve stimulation

### Declarations

## Conflict of interest:

MV has patents related to neuromodulation held by UCLA with minor shares in NeuCures Inc. and Anumana Inc.

## FUNDING

This study was funded by NIHR01HL148190 and NIHR01HL70626 to MV and NWO Rubicon to VvW.

## ACKNOWLEDGEMENTS

The authors are grateful for the help of the University of California, Los Angeles (UCLA) Proteome Research Center.

## Data Availability

Plasma mass spectrometry data has been deposited to the ProteomeXchange Consortium via the PRIDE partner repository. Separate dataset files used for analyses are available in Dataset Files 1 and 2. All other data and metadata are available from the corresponding author upon reasonable request.

## References

1. van Weperen, V. Y. H., Ripplinger, C. M. & Vaseghi, M. Autonomic control of ventricular function in health and disease: current state of the art. *Clin Auton Res* **33**, 1-27 (2023). https://doi.org/10.1007/s10286-023-00948-8
2. Vaseghi, M. & Shivkumar, K. The role of the autonomic nervous system in sudden cardiac death. *Prog Cardiovasc Dis* **50**, 404-419 (2008). https://doi.org/10.1016/j.pcad.2008.01.003
3. Ajijola, O. A. *et al.* Inflammation, oxidative stress, and glial cell activation characterize stellate ganglia from humans with electrical storm. *JCI Insight* **2**, e94715 (2017). https://doi.org/10.1172/jci.insight.94715
4. Ajijola, O. A. *et al.* Extracardiac neural remodeling in humans with cardiomyopathy. *Circ Arrhythm Electrophysiol* **5**, 1010-1116 (2012). https://doi.org/10.1161/CIRCEP.112.972836
5. Wang, H. J., Rozanski, G. J. & Zucker, I. H. Cardiac sympathetic afferent reflex control of cardiac function in normal and chronic heart failure states. *J Physiol* **595**, 2519-2534 (2017). https://doi.org/10.1113/JP273764
6. Wang, H. J., Wang, W., Cornish, K. G., Rozanski, G. J. & Zucker, I. H. Cardiac sympathetic afferent denervation attenuates cardiac remodeling and improves cardiovascular dysfunction in rats with heart failure. *Hypertension* **64**, 745-755 (2014). https://doi.org/10.1161/HYPERTENSIONAHA.114.03699
7. Chen, W. W. *et al.* Cardiac sympathetic afferent reflex and its implications for sympathetic activation in chronic heart failure and hypertension. *Acta Physiol (Oxf)* **213**, 778-794 (2015). https://doi.org/10.1111/apha.12447
8. Zhu, G. Q., Zucker, I. H. & Wang, W. Central AT1 receptors are involved in the enhanced cardiac sympathetic afferent reflex in rats with chronic heart failure. *Basic Res Cardiol* **97**, 320-326 (2002). https://doi.org/10.1007/s00395-002-0353-z
9. Wang, W. Z., Gao, L., Wang, H. J., Zucker, I. H. & Wang, W. Interaction between cardiac sympathetic afferent reflex and chemoreflex is mediated by the NTS AT1 receptors in heart failure. *Am J Physiol Heart Circ Physiol* **295**, H1216-H1226 (2008). https://doi.org/10.1152/ajpheart.00557.2008
10. Vaseghi, M., Lux, R. L., Mahajan, A. & Shivkumar, K. Sympathetic stimulation increases dispersion of repolarization in humans with myocardial infarction. *Am J Physiol Heart Circ Physiol* **302**, H1838-1846 (2012). https://doi.org/10.1152/ajpheart.01106.2011
11. Ben-David, J. & Zipes, D. P. Differential response to right and left ansae subclaviae stimulation of early afterdepolarizations and ventricular tachycardia induced by cesium in dogs. *Circulation* **78**, 1241-1250 (1988). https://doi.org/10.1161/01.cir.78.5.1241
12. Priori, S. G., Mantica, M. & Schwartz, P. J. Delayed afterdepolarizations elicited in vivo by left stellate ganglion stimulation. *Circulation* **78**, 178-185 (1988). https://doi.org/10.1161/01.cir.78.1.178
13. Opthof, T. *et al.* Dispersion of refractoriness in normal and ischaemic canine ventricle: effects of sympathetic stimulation. *Cardiovasc Res* **27**, 1954-1960 (1993).

https://doi.org/10.1093/cvr/27.11.1954

14. Olivas, A. *et al.* Myocardial Infarction Causes Transient Cholinergic Transdifferentiation of Cardiac Sympathetic Nerves via gp130. *J Neurosci* **36**, 479-488 (2016). https://doi.org/10.1523/JNEUROSCI.3556-15.2016
15. Devarajan, A. *et al.* Myocardial infarction causes sex-dependent dysfunction in vagal sensory glutamatergic neurotransmission that is mitigated by 17beta-Estradiol. *JCI Insight* **9**, e181042 (2024). https://doi.org/10.1172/jci.insight.181042
16. Gao, C. *et al.* Inflammatory and apoptotic remodeling in autonomic nervous system following myocardial infarction. *PLoS One* **12**, e0177750 (2017). https://doi.org/10.1371/journal.pone.0177750
17. Stueber, T. *et al.* Differential cytotoxicity and intracellular calcium-signalling following activation of the calcium-permeable ion channels TRPV1 and TRPA1. *Cell Calcium* **68**, 34-44 (2017). https://doi.org/10.1016/j.ceca.2017.10.003
18. Armour, J. A. & Ardell, J. L. *Basic and clinical neurocardiology*. (Oxford University Press, 2004).
19. Maggi, C. A. Tachykinins and calcitonin gene-related peptide (CGRP) as co-transmitters released from peripheral endings of sensory nerves. *Prog Neurobiol* **45**, 1-98 (1995). https://doi.org/10.1016/0301-0082(94)e0017-b
20. Gibson, S. J. *et al.* Calcitonin gene-related peptide immunoreactivity in the spinal cord of man and of eight other species. *J Neurosci* **4**, 3101-3111 (1984). https://doi.org/10.1523/JNEUROSCI.04-12-03101.1984
21. Schaible, H. G. in *Encyclopedia of Pain* (eds G.F. Gebhart & R.F. Schmidt) (Springer, 2013).
22. La Rovere, M. T., Bigger, J. T., Jr., Marcus, F. I., Mortara, A. & Schwartz, P. J. Baroreflex sensitivity and heart-rate variability in prediction of total cardiac mortality after myocardial infarction. ATRAMI (Autonomic Tone and Reflexes After Myocardial Infarction) Investigators. *Lancet* **351**, 478-484 (1998). https://doi.org/10.1016/s0140-6736(97)11144-8
23. Mortara, A. *et al.* Arterial baroreflex modulation of heart rate in chronic heart failure: clinical and hemodynamic correlates and prognostic implications. *Circulation* **96**, 3450-3458 (1997). https://doi.org/10.1161/01.cir.96.10.3450
24. Schwartz, P. J. *et al.* Autonomic mechanisms and sudden death. New insights from analysis of baroreceptor reflexes in conscious dogs with and without a myocardial infarction. *Circulation* **78**, 969-979 (1988). https://doi.org/10.1161/01.cir.78.4.969
25. Rajendran, P. S. *et al.* Myocardial infarction induces structural and functional remodelling of the intrinsic cardiac nervous system. *J Physiol* **594**, 321-341 (2016). https://doi.org/10.1113/JP271165
26. Ardell, J. L. & Armour, J. A. Neurocardiology: Structure-Based Function. *Compr Physiol* **6**, 1635-1653 (2016). https://doi.org/10.1002/cphy.c150046
27. Giannino, G. *et al.* The Intrinsic Cardiac Nervous System: From Pathophysiology to Therapeutic Implications. *Biology (Basel)* **13** (2024). https://doi.org/10.3390/biology13020105

28. Beaumont, E. *et al.* Network interactions within the canine intrinsic cardiac nervous system: implications for reflex control of regional cardiac function. *J Physiol* **591**, 4515-4533 (2013). https://doi.org/10.1113/jphysiol.2013.259382
29. Hoang, J. D. *et al.* Antiarrhythmic Mechanisms of Epidural Blockade After Myocardial Infarction. *Circ Res* **135**, e57-e75 (2024). https://doi.org/10.1161/CIRCRESAHA.123.324058
30. Vaseghi, M. *et al.* Parasympathetic dysfunction and antiarrhythmic effect of vagal nerve stimulation following myocardial infarction. *JCI Insight* **2**, e86715 (2017). https://doi.org/10.1172/jci.insight.86715
31. Yagishita, D. *et al.* Sympathetic nerve stimulation, not circulating norepinephrine, modulates T-peak to T-end interval by increasing global dispersion of repolarization. *Circ Arrhythm Electrophysiol* **8**, 174-185 (2015). https://doi.org/10.1161/CIRCEP.114.002195
32. Gardner, R. T. *et al.* Targeting protein tyrosine phosphatase sigma after myocardial infarction restores cardiac sympathetic innervation and prevents arrhythmias. *Nat Commun* **6**, 6235 (2015). https://doi.org/10.1038/ncomms7235
33. Cao, J. M. *et al.* Relationship between regional cardiac hyperinnervation and ventricular arrhythmia. *Circulation* **101**, 1960-1969 (2000). https://doi.org/10.1161/01.cir.101.16.1960
34. Rubart, M. & Zipes, D. P. Mechanisms of sudden cardiac death. *J Clin Invest* **115**, 2305-2315 (2005). https://doi.org/10.1172/JCI26381
35. Warner, M. R., Wisler, P. L., Hodges, T. D., Watanabe, A. M. & Zipes, D. P. Mechanisms of denervation supersensitivity in regionally denervated canine hearts. *Am J Physiol* **264**, H815-820 (1993). https://doi.org/10.1152/ajpheart.1993.264.3.H815
36. Kammerling, J. J. *et al.* Denervation supersensitivity of refractoriness in noninfarcted areas apical to transmural myocardial infarction. *Circulation* **76**, 383-393 (1987). https://doi.org/10.1161/01.cir.76.2.383
37. Inoue, H. & Zipes, D. P. Time course of denervation of efferent sympathetic and vagal nerves after occlusion of the coronary artery in the canine heart. *Circ Res* **62**, 1111-1120 (1988). https://doi.org/10.1161/01.res.62.6.1111
38. Prabhu, S. D. & Frangogiannis, N. G. The Biological Basis for Cardiac Repair After Myocardial Infarction: From Inflammation to Fibrosis. *Circ Res* **119**, 91-112 (2016). https://doi.org/10.1161/CIRCRESAHA.116.303577
39. Wang, M. *et al.* Increased inflammation promotes ventricular arrhythmia through aggravating left stellate ganglion remodeling in a canine ischemia model. *Int J Cardiol* **248**, 286-293 (2017). https://doi.org/10.1016/j.ijcard.2017.08.011
40. Fischer, A. *et al.* ZAP70: a master regulator of adaptive immunity. *Semin Immunopathol* **32**, 107-116 (2010). https://doi.org/10.1007/s00281-010-0196-x
41. Qureshi, N., Morrison, D. C. & Reis, J. Proteasome protease mediated regulation of cytokine induction and inflammation. *Biochim Biophys Acta* **1823**, 2087-2093 (2012). https://doi.org/10.1016/j.bbamcr.2012.06.016

42. Liu, H., Yu, S., Xu, W. & Xu, J. Enhancement of 26S proteasome functionality connects oxidative stress and vascular endothelial inflammatory response in diabetes mellitus. *Arterioscler Thromb Vasc Biol* **32**, 2131-2140 (2012). https://doi.org/10.1161/ATVBAHA.112.253385
43. Neurath, M. F. & Berg, L. J. VAV1 as a putative therapeutic target in autoimmune and chronic inflammatory diseases. *Trends Immunol* **45**, 580-596 (2024). https://doi.org/10.1016/j.it.2024.06.004
44. Gilbert, C. J., Longenecker, J. Z. & Accornero, F. ERK1/2: An Integrator of Signals That Alters Cardiac Homeostasis and Growth. *Biology (Basel)* **10** (2021). https://doi.org/10.3390/biology10040346
45. Marchlinski, F. E., Callans, D. J., Gottlieb, C. D. & Zado, E. Linear ablation lesions for control of unmappable ventricular tachycardia in patients with ischemic and nonischemic cardiomyopathy. *Circulation* **101**, 1288-1296 (2000). https://doi.org/10.1161/01.cir.101.11.1288
46. Hamdan, M. H. *et al.* Biventricular pacing decreases sympathetic activity compared with right ventricular pacing in patients with depressed ejection fraction. *Circulation* **102**, 1027-1032 (2000). https://doi.org/10.1161/01.cir.102.9.1027
47. Taylor, J. A., Morillo, C. A., Eckberg, D. L. & Ellenbogen, K. A. Higher sympathetic nerve activity during ventricular (VVI) than during dual-chamber (DDD) pacing. *J Am Coll Cardiol* **28**, 1753-1758 (1996). https://doi.org/10.1016/s0735-1097(96)00389-0
48. Do, D. H. *et al.* Thoracic Epidural Anesthesia Can Be Effective for the Short-Term Management of Ventricular Tachycardia Storm. *J Am Heart Assoc* **6**, e007080 (2017). https://doi.org/10.1161/JAHA.117.007080
49. Kang, K. W. Successful neural modulation of bedside modified thoracic epidural anesthesia for ventricular tachycardia electrical storm. *Acute Crit Care* **39**, 643-646 (2022). https://doi.org/10.4266/acc.2021.01683
50. Salavatian, S. *et al.* Myocardial infarction reduces cardiac nociceptive neurotransmission through the vagal ganglia. *JCI Insight* **7** (2022). https://doi.org/10.1172/jci.insight.155747
51. Nakamura, K. *et al.* Pathological effects of chronic myocardial infarction on peripheral neurons mediating cardiac neurotransmission. *Auton Neurosci* **197**, 34-40 (2016). https://doi.org/10.1016/j.autneu.2016.05.001
52. Cao, J. M. *et al.* Nerve sprouting and sudden cardiac death. *Circ Res* **86**, 816-821 (2000). https://doi.org/10.1161/01.res.86.7.816
53. Shanks, J., de Morais, S. D. B., Gao, L., Zucker, I. H. & Wang, H. J. TRPV1 (Transient Receptor Potential Vanilloid 1) Cardiac Spinal Afferents Contribute to Hypertension in Spontaneous Hypertensive Rat. *Hypertension* **74**, 910-920 (2019). https://doi.org/10.1161/HYPERTENSIONAHA.119.13285
54. Wang, D. *et al.* Focal selective chemo-ablation of spinal cardiac afferent nerve by resiniferatoxin protects the heart from pressure overload-induced hypertrophy. *Biomed Pharmacother* **109**, 377-385 (2019). https://doi.org/10.1016/j.biopha.2018.10.156

55. Zhu, G. Q. *et al.* Enhanced cardiac sympathetic afferent reflex involved in sympathetic overactivity in renovascular hypertensive rats. *Exp Physiol* **94**, 785-794 (2009). https://doi.org/10.1113/expphysiol.2008.046565
56. Gao, L., Schultz, H. D., Patel, K. P., Zucker, I. H. & Wang, W. Augmented input from cardiac sympathetic afferents inhibits baroreflex in rats with heart failure. *Hypertension* **45**, 1173-1181 (2005). https://doi.org/10.1161/01.HYP.0000168056.66981.c2
57. Schwartz, P. J., Pagani, M., Lombardi, F., Malliani, A. & Brown, A. M. A cardiocardiac sympathovagal reflex in the cat. *Circ Res* **32**, 215-220 (1973). https://doi.org/10.1161/01.res.32.2.215
58. Zahner, M. R., Li, D. P., Chen, S. R. & Pan, H. L. Cardiac vanilloid receptor 1-expressing afferent nerves and their role in the cardiogenic sympathetic reflex in rats. *The Journal of Physiology* **551**, 515-523 (2003). https://doi.org/10.1113/jphysiol.2003.048207
59. Pan, H. L., Chen, S. R., Scicli, G. M. & Carretero, O. A. Cardiac interstitial bradykinin release during ischemia is enhanced by ischemic preconditioning. *Am J Physiol Heart Circ Physiol* **279**, H116-121 (2000). https://doi.org/10.1152/ajpheart.2000.279.1.H116
60. Ide, R., Saiki, C., Makino, M. & Matsumoto, S. TRPV1 receptor expression in cardiac vagal afferent neurons of infant rats. *Neurosci Lett* **507**, 67-71 (2012). https://doi.org/10.1016/j.neulet.2011.11.055
61. Mohammed, M., Madden, C. J., Andresen, M. C. & Morrison, S. F. Activation of TRPV1 in nucleus tractus solitarius reduces brown adipose tissue thermogenesis, arterial pressure, and heart rate. *Am J Physiol Regul Integr Comp Physiol* **315**, R134-R143 (2018). https://doi.org/10.1152/ajpregu.00049.2018
62. Peng, C. *et al.* Neuroimmune modulation mediated by IL-6: A potential target for the treatment of ischemia-induced ventricular arrhythmias. *Heart Rhythm* **21**, 610-619 (2024). https://doi.org/10.1016/j.hrthm.2023.12.020
63. Wu, C. *et al.* Spinal cord astrocytes regulate myocardial ischemia-reperfusion injury. *Basic Res Cardiol* **117**, 56 (2022). https://doi.org/10.1007/s00395-022-00968-x
64. Deng, J. *et al.* The effects of interleukin 17A on left stellate ganglion remodeling are mediated by neuroimmune communication in normal structural hearts. *Int J Cardiol* **279**, 64-71 (2019). https://doi.org/10.1016/j.ijcard.2019.01.010
65. Padfield, G. J. *et al.* Cardiovascular effects of tumour necrosis factor alpha antagonism in patients with acute myocardial infarction: a first in human study. *Heart* **99**, 1330-1335 (2013). https://doi.org/10.1136/heartjnl-2013-303648
66. Mann, D. L. *et al.* Targeted anticytokine therapy in patients with chronic heart failure: results of the Randomized Etanercept Worldwide Evaluation (RENEWAL). *Circulation* **109**, 1594-1602 (2004). https://doi.org/10.1161/01.CIR.0000124490.27666.B2
67. Chung, E. S. *et al.* Randomized, double-blind, placebo-controlled, pilot trial of infliximab, a chimeric monoclonal antibody to tumor necrosis factor-alpha, in patients with moderate-to-severe heart failure: results of the anti-TNF Therapy Against Congestive Heart Failure (ATTACH) trial. *Circulation* **107**, 3133-3140 (2003). https://doi.org/10.1161/01.CIR.0000077913.60364.D2

68. Huang, S. *et al.* Distinct roles of myofibroblast-specific Smad2 and Smad3 signaling in repair and remodeling of the infarcted heart. *J Mol Cell Cardiol* **132**, 84-97 (2019). https://doi.org/10.1016/j.yjmcc.2019.05.006
69. Kong, P. *et al.* Opposing Actions of Fibroblast and Cardiomyocyte Smad3 Signaling in the Infarcted Myocardium. *Circulation* **137**, 707-724 (2018). https://doi.org/10.1161/CIRCULATIONAHA.117.029622
70. Hanna, A., Humeres, C. & Frangogiannis, N. G. The role of Smad signaling cascades in cardiac fibrosis. *Cell Signal* **77**, 109826 (2021). https://doi.org/10.1016/j.cellsig.2020.109826
71. Kettenmann, H., Hanisch, U. K., Noda, M. & Verkhratsky, A. Physiology of microglia. *Physiol Rev* **91**, 461-553 (2011). https://doi.org/10.1152/physrev.00011.2010
72. Shen, J., Bian, N., Zhao, L. & Wei, J. The role of T-lymphocytes in central nervous system diseases. *Brain Res Bull* **209**, 110904 (2024). https://doi.org/10.1016/j.brainresbull.2024.110904
73. Salavatian, S. *et al.* Myocardial infarction reduces cardiac nociceptive neurotransmission through the vagal ganglia. *JCI Insight* **7**, e155747 (2022). https://doi.org/10.1172/jci.insight.155747
74. Ping Dai, R., Ping He, B., Thameem Dheen, S. & Tay, S. S. Acute cardiac injury induces glial cell response and activates extracellular signaling-regulated kinase-1 and -2 in the spinal cord of Wistar rats. *Neurosci Lett* **366**, 34-38 (2004). https://doi.org/10.1016/j.neulet.2004.05.018
75. Zipes, D. P. & Wellens, H. J. Sudden cardiac death. *Circulation* **98**, 2334-2351 (1998). https://doi.org/10.1161/01.cir.98.21.2334
76. Shen, M. J. & Zipes, D. P. Role of the autonomic nervous system in modulating cardiac arrhythmias. *Circ Res* **114**, 1004-1021 (2014). https://doi.org/10.1161/CIRCRESAHA.113.302549
77. Fallavollita, J. A. *et al.* Regional myocardial sympathetic denervation predicts the risk of sudden cardiac arrest in ischemic cardiomyopathy. *J Am Coll Cardiol* **63**, 141-149 (2014). https://doi.org/10.1016/j.jacc.2013.07.096
78. Taggart, P. *et al.* Effect of adrenergic stimulation on action potential duration restitution in humans. *Circulation* **107**, 285-289 (2003). https://doi.org/10.1161/01.cir.0000044941.13346.74
79. Taggart, P., Sutton, P., Lab, M., Dean, J. & Harrison, F. Interplay between adrenaline and interbeat interval on ventricular repolarisation in intact heart in vivo. *Cardiovasc Res* **24**, 884-895 (1990). https://doi.org/10.1093/cvr/24.11.884
80. Huang, B., Qin, D. & El-Sherif, N. Early down-regulation of K+ channel genes and currents in the postinfarction heart. *J Cardiovasc Electrophysiol* **11**, 1252-1261 (2000). https://doi.org/10.1046/j.1540-8167.2000.01252.x
81. Patterson, E. *et al.* Sodium-calcium exchange initiated by the Ca2+ transient: an arrhythmia trigger within pulmonary veins. *J Am Coll Cardiol* **47**, 1196-1206 (2006). https://doi.org/10.1016/j.jacc.2005.12.023

82. Do, D. H. *et al.* Thoracic Epidural Anesthesia Can Be Effective for the Short-Term Management of Ventricular Tachycardia Storm. *J Am Heart Assoc* **6** (2017). https://doi.org/10.1161/JAHA.117.007080

83. Vaseghi, M. *et al.* Cardiac sympathetic denervation in patients with refractory ventricular arrhythmias or electrical storm: intermediate and long-term follow-up. *Heart Rhythm* **11**, 360-366 (2014). https://doi.org/10.1016/j.hrthm.2013.11.028

84. Irie, T. *et al.* Cardiac sympathetic innervation via middle cervical and stellate ganglia and antiarrhythmic mechanism of bilateral stellectomy. *Am J Physiol Heart Circ Physiol* **312**, 392-405 (2017). https://doi.org/10.1152/ajpheart.00644.2016

85. Baker, D. G., Coleridge, H. M., Coleridge, J. C. & Nerdrum, T. Search for a cardiac nociceptor: stimulation by bradykinin of sympathetic afferent nerve endings in the heart of the cat. *J Physiol* **306**, 519-536 (1980). https://doi.org/10.1113/jphysiol.1980.sp013412

86. Nerdrum, T., Baker, D. G., Coleridge, H. M. & Coleridge, J. C. Interaction of bradykinin and prostaglandin E1 on cardiac pressor reflex and sympathetic afferents. *Am J Physiol* **250**, R815-822 (1986). https://doi.org/10.1152/ajpregu.1986.250.5.R815

87. Yamaguchi, T. *et al.* Thoracic Dorsal Root Ganglion Application of Resiniferatoxin Reduces Myocardial Ischemia-Induced Ventricular Arrhythmias. *Biomedicines* **11** (2023). https://doi.org/10.3390/biomedicines11102720

88. Hoang, J. D. *et al.* Proarrhythmic Effects of Sympathetic Activation Are Mitigated by Vagal Nerve Stimulation in Infarcted Hearts. *JACC Clin Electrophysiol* **8**, 513-525 (2022). https://doi.org/10.1016/j.jacep.2022.01.018

89. Vaseghi, M. *et al.* Parasympathetic dysfunction and antiarrhythmic effect of vagal nerve stimulation following myocardial infarction. *JCI Insight* **2** (2017). https://doi.org/10.1172/jci.insight.86715

90. Haws, C. W. & Lux, R. L. Correlation between in vivo transmembrane action potential durations and activation-recovery intervals from electrograms. Effects of interventions that alter repolarization time. *Circulation* **81**, 281-288 (1990). https://doi.org/10.1161/01.cir.81.1.281

91. Smyth, H. S., Sleight, P. & Pickering, G. W. Reflex regulation of arterial pressure during sleep in man. A quantitative method of assessing baroreflex sensitivity. *Circ Res* **24**, 109-121 (1969). https://doi.org/10.1161/01.res.24.1.109

92. Salavatian, S. *et al.* Vagal stimulation targets select populations of intrinsic cardiac neurons to control neurally induced atrial fibrillation. *Am J Physiol Heart Circ Physiol* **311**, H1311-H1320 (2016). https://doi.org/10.1152/ajpheart.00443.2016

93. Shin, H. C., Aggarwal, V., Acharya, S., Schieber, M. H. & Thakor, N. V. Neural decoding of finger movements using Skellam-based maximum-likelihood decoding. *IEEE Trans Biomed Eng* **57**, 754-760 (2010). https://doi.org/10.1109/TBME.2009.2020791

94. Wellens, H. J., Brugada, P. & Stevenson, W. G. Programmed electrical stimulation of the heart in patients with life-threatening ventricular arrhythmias: what is the significance of induced arrhythmias and what is the correct stimulation protocol? *Circulation* **72**, 1-7 (1985). https://doi.org/10.1161/01.cir.72.1.1

95. Meng, J. *et al.* Activation of TRPV1 mediates calcitonin gene-related peptide release, which excites trigeminal sensory neurons and is attenuated by a retargeted botulinum toxin with anti-nociceptive potential. *J Neurosci* **29**, 4981-4992 (2009). https://doi.org/10.1523/JNEUROSCI.5490-08.2009
96. Wu, C. C. *et al.* Mag-Net: Rapid enrichment of membrane-bound particles enables high coverage quantitative analysis of the plasma proteome. *bioRxiv* (2024). https://doi.org/10.1101/2023.06.10.544439
97. Stewart, H. I. *et al.* Parallelized Acquisition of Orbitrap and Astral Analyzers Enables High-Throughput Quantitative Analysis. *Anal Chem* **95**, 15656-15664 (2023). https://doi.org/10.1021/acs.analchem.3c02856
98. Guzman, U. H. *et al.* Ultra-fast label-free quantification and comprehensive proteome coverage with narrow-window data-independent acquisition. *Nat Biotechnol* **42**, 1855-1866 (2024). https://doi.org/10.1038/s41587-023-02099-7
99. Demichev, V., Messner, C. B., Vernardis, S. I., Lilley, K. S. & Ralser, M. DIA-NN: neural networks and interference correction enable deep proteome coverage in high throughput. *Nat Methods* **17**, 41-44 (2020). https://doi.org/10.1038/s41592-019-0638-x
100. Hsiao, Y. *et al.* Analysis and Visualization of Quantitative Proteomics Data Using FragPipe-Analyst. *J Proteome Res* **23**, 4303-4315 (2024). https://doi.org/10.1021/acs.jproteome.4c00294
101. Zimmermann, M. T., Kabat, B., Grill, D. E., Kennedy, R. B. & Poland, G. A. RITAN: rapid integration of term annotation and network resources. *PeerJ* **7**, e6994 (2019). https://doi.org/10.7717/peerj.6994

## Figures

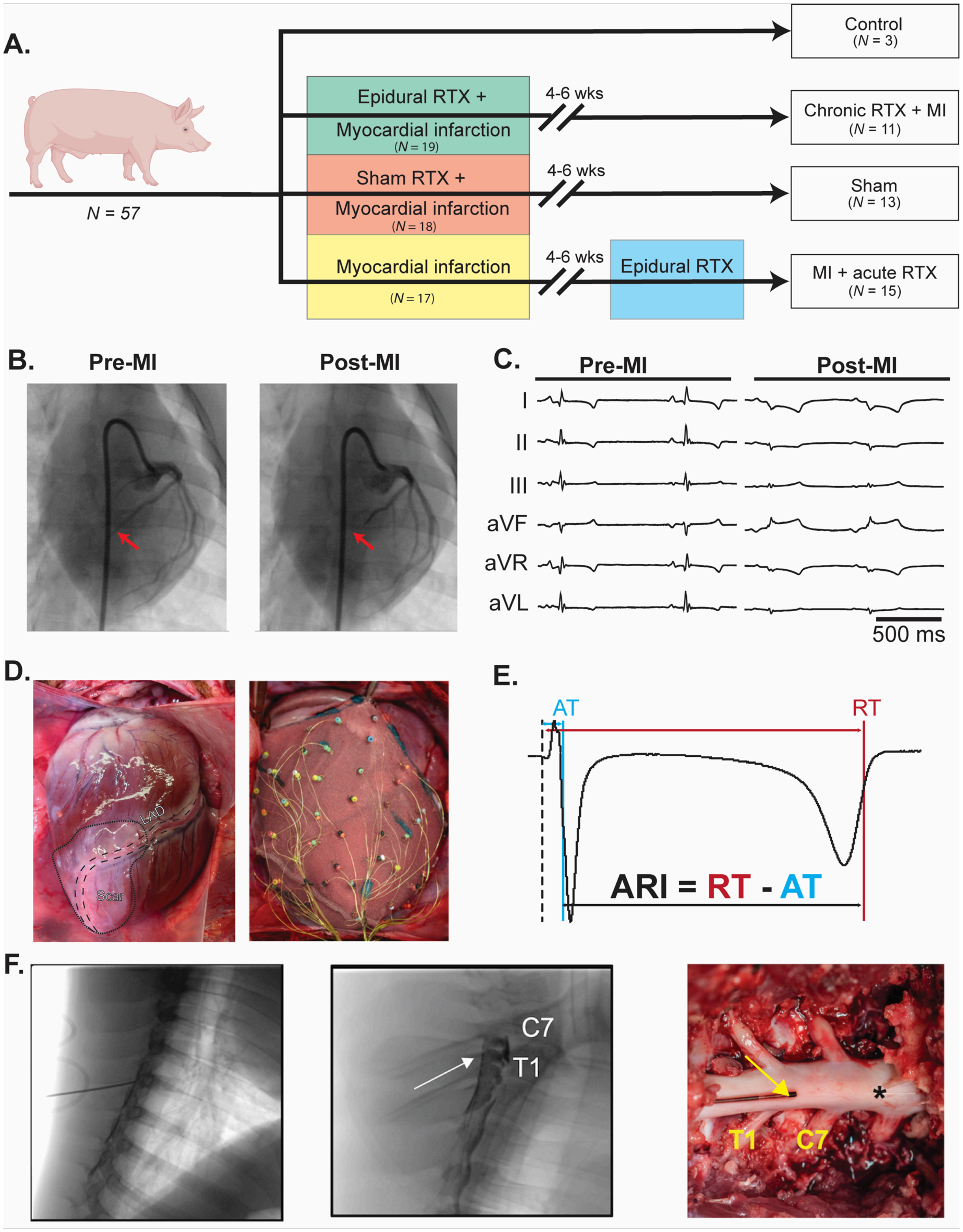

Figure 1

*Experimental protocol.* **(A)** Schematic representation of the experimental groups and their respective interventions. **(B)** Percutaneous creation of myocardial infarction (MI) via injection of microspheres in the distal left anterior descending (LAD) coronary artery after percutaneous angioplasty balloon inflation. Following injection, flow is significantly decreased in the mid and distal LAD (red arrows) and **(C)** ST-segment elevation is observed on the surface ECG. **(D)** At the time of terminal studies, a 56-electrode sock was placed around the ventricles to record local unipolar electrograms, from which **(E)** repolarization time (RT) and activation time (AT) were measured and activation recovery interval (ARI) calculated in all infarcted animals. **(F)** For epidural injections, an epidural catheter (white arrow) was advanced to the C7-T1 dorsal epidural space and position confirmed by contrast injection under fluoroscopic guidance. In terminal procedures involving acute epidural RTX injections, catheter position was secondarily confirmed at the end of experiments by cut down to the epidural space and removal of epidural fat. Asterisk placed at cranial aspect of the spinal cord.

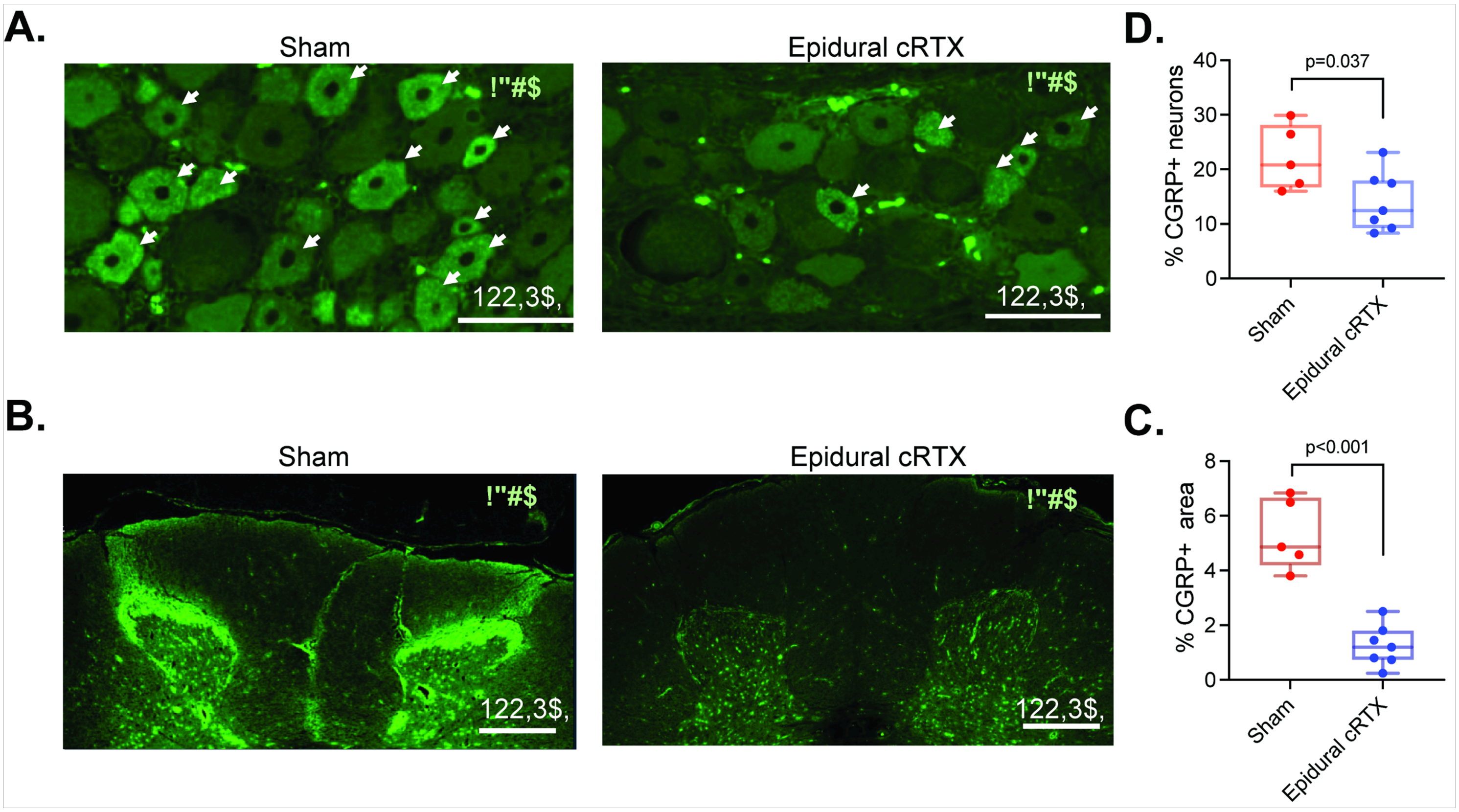

### Figure 2

***Immunohistochemical confirmation of successful ablation of cardiac spinal nociceptive neurons and fibers.*** **(A)** Representative images of calcitonin gene-related peptide (CGRP) immunostaining of the dorsal root ganglia from a chronic sham and an epidural cRTX animal. **(B)** A significant decrease in CGRP expressing neurons in the dorsal root ganglia of epidural cRTX animals (*n*=7) *vs* sham animals (*n*=5) is observed. **(C)** Representative images of CGRP immunostaining in the dorsal horn from chronic sham and epidural cRTX animals. **(D)** A significant decrease in dorsal horn CGRP expression in epidural cRTX animals (*n*=7) *vs* sham animals (*n*=5) is observed, confirming successful chemoablation of spinal nociceptive cardiac afferents in animals that received RTX. Differences between sham and epidural cRTX animals were assessed using the unpaired Student’s t-test.

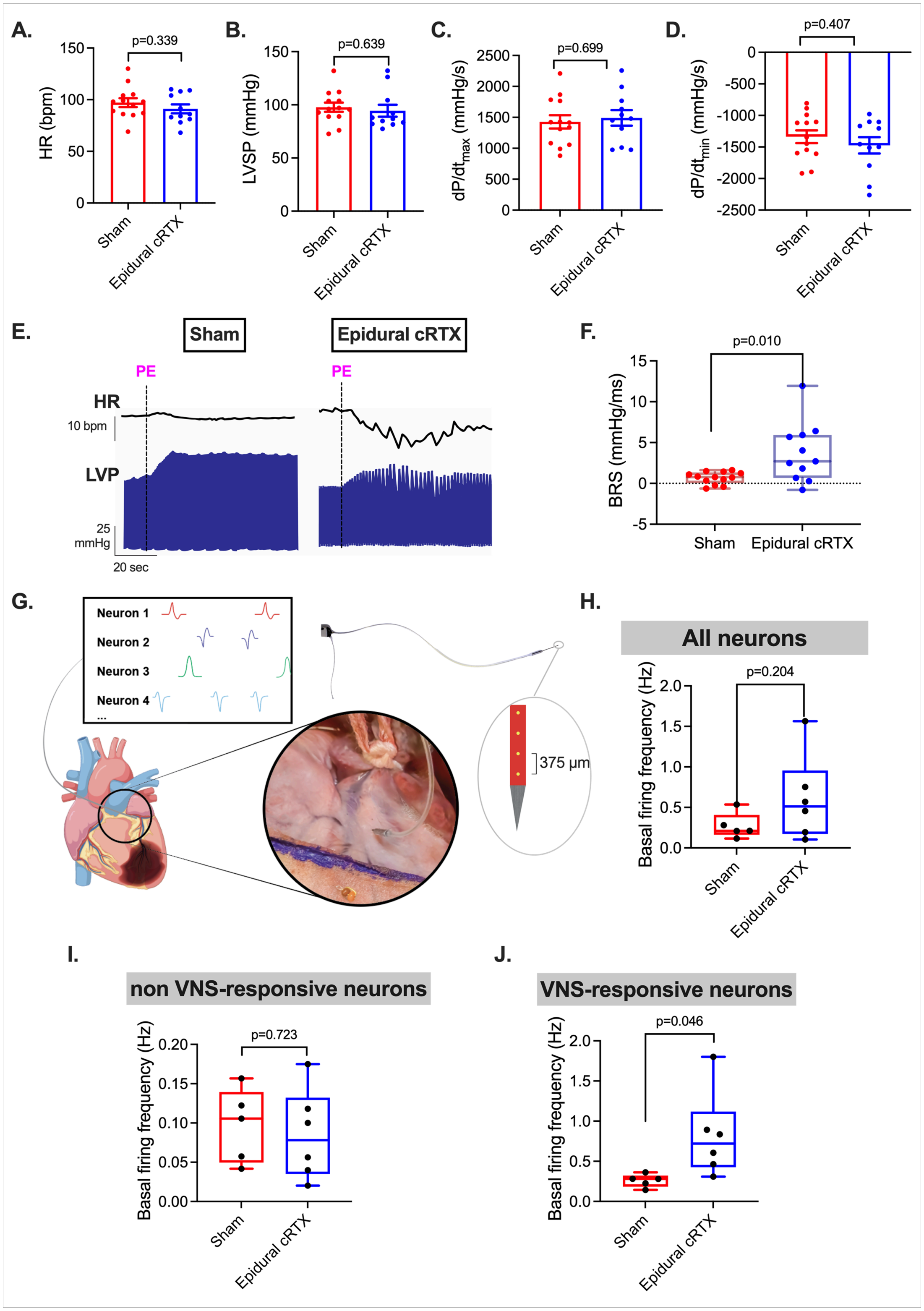

### Figure 3

***Effects of nociceptive afferent ablation prior to MI on cardiac hemodynamics and vagal tone four to six weeks after MI.*** **(A-D)** Baseline hemodynamic parameters were not different between sham (*n*=13) and epidural cRTX (*n*=11) animals. **(E)** Example of phenylephrine-evoked baroreflex responses in a sham and epidural cRTX animal. **(F)** Baroreflex sensitivity (BRS) was significantly improved in epidural cRTX animals (*n*=11) compared to sham animals (*n*=13). **(G)** Schematic image of ventral interventricular ganglionated plexus (VIVGP) neural recordings and identification of VIVGP neurons. A magnified image of the neural recording electrode in a VIVGP is shown. The left atrial appendage is being retracted to fully expose the VIVGP. A picture of the neural probe and a schematic of the tip with the most distal 4 recording electrodes is shown. There was no difference in basal firing frequency of all **(H)** or non-VNS-responsive **(I)** neurons. **(J)** However, the basal firing frequency of VNS-responsive neurons (*e.g.*, parasympathetic postganglionic neurons) was significantly higher in epidural cRTX animals ($n$=6) compared to sham animals ($n$=5). Differences in HR, LVSP, $dP/dt_{max}$, $dP/dt_{min}$, and neuronal firing between sham and epidural cRTX animals were assessed using the unpaired Student's $t$-test, and BRS was compared by the Mann-Whitney test. PE: Phenylephrine

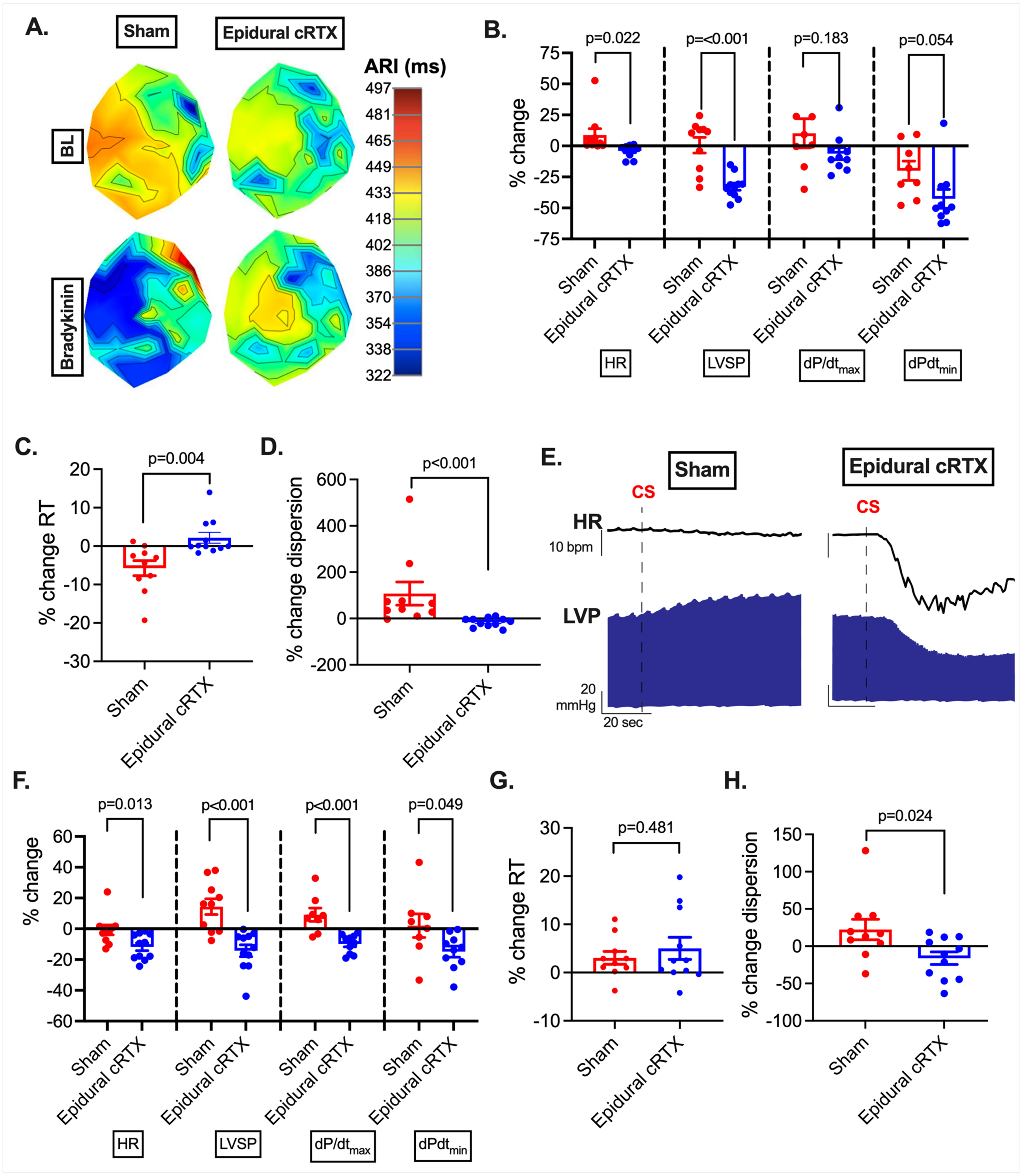

### Figure 4

***Effects of spinal afferent ablation prior to MI on reflex-evoked cardiac function four to six weeks after MI.*** **(A)** Representative 2D-map of electrophysiological changes evoked by epicardial application of bradykinin in a sham (left) and an epidural cRTX animal (right). **(B-D)** Quantified hemodynamic and electrophysiological responses to epicardial application of bradykinin in sham (*n*=10) and epidural cRTX (*n*=11) animals are shown. **(E)** Representation of hemodynamic changes evoked by epicardial application of capsaicin in a sham (upper) and an epidural cRTX animal (lower). **(F-H)** Quantified hemodynamic and electrophysiological changes in repolarization time (RT) and dispersion of RT upon epicardial application of capsaicin in sham (*n*=10) and epidural cRTX (*n*=11) animals. Differences in HR, LVSP, $dP/dt_{max}$, $dP/dt_{min}$, RT and dispersion responses upon bradykinin and capsaicin administration between sham and epidural cRTX animals were assessed using the unpaired Student's *t*-test. CS: Capsaicin.

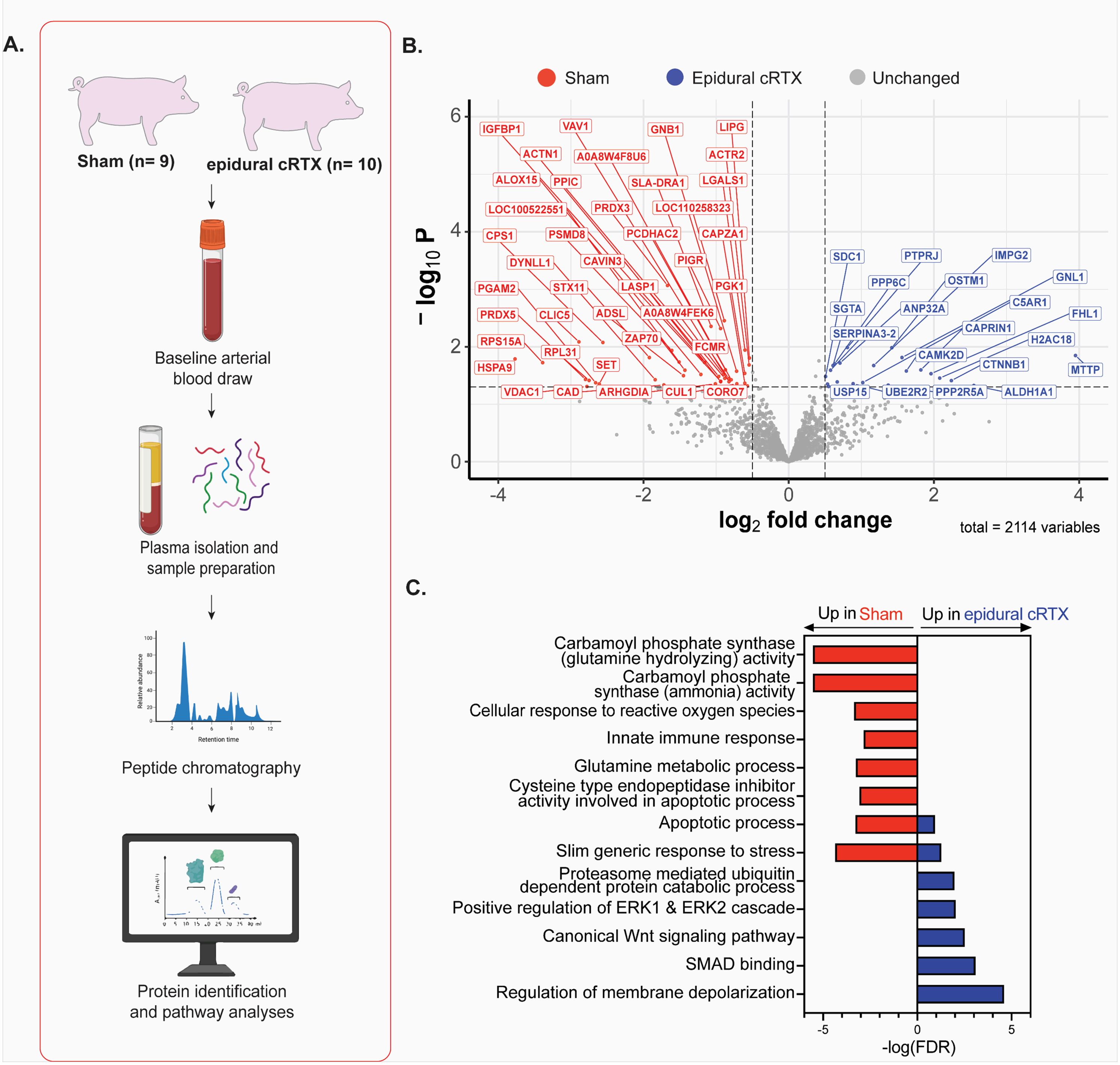

### Figure 5

***Proteomic analyses demonstrate a decrease in inflammatory and stress response pathways in infarcted animals with RTX treatment prior to MI (epidural cRTX) compared to untreated, infarcted animals (sham)***

***four to six weeks post infarction.*** **(A)** Schematic representation of plasma protein isolation to proteomic data analyses. **(B)** Volcano plot showing the differentially expressed proteins in sham and epidural cRTX animals. **(C)** Proteomic pathway analyses conducted on expression data against the GO database show a significant decrease in oxidative stress and inflammatory pathways in epidural cRTX animals, compared to sham animals. Pathways associated with ERK1 and ERK2 cascades were upregulated in epidural cRTX animals, which have been described to be fundamental for cardiomyocyte homeostasis and stress responses. Differentially expressed proteins were identified using a two-sided Student's t-test. Pathway analysis was performed against the Gene Ontology (GO) database using Rapid Integration of Term Annotation and Network. *n*=9 for MI, *n*=10 for epidural cRTX.

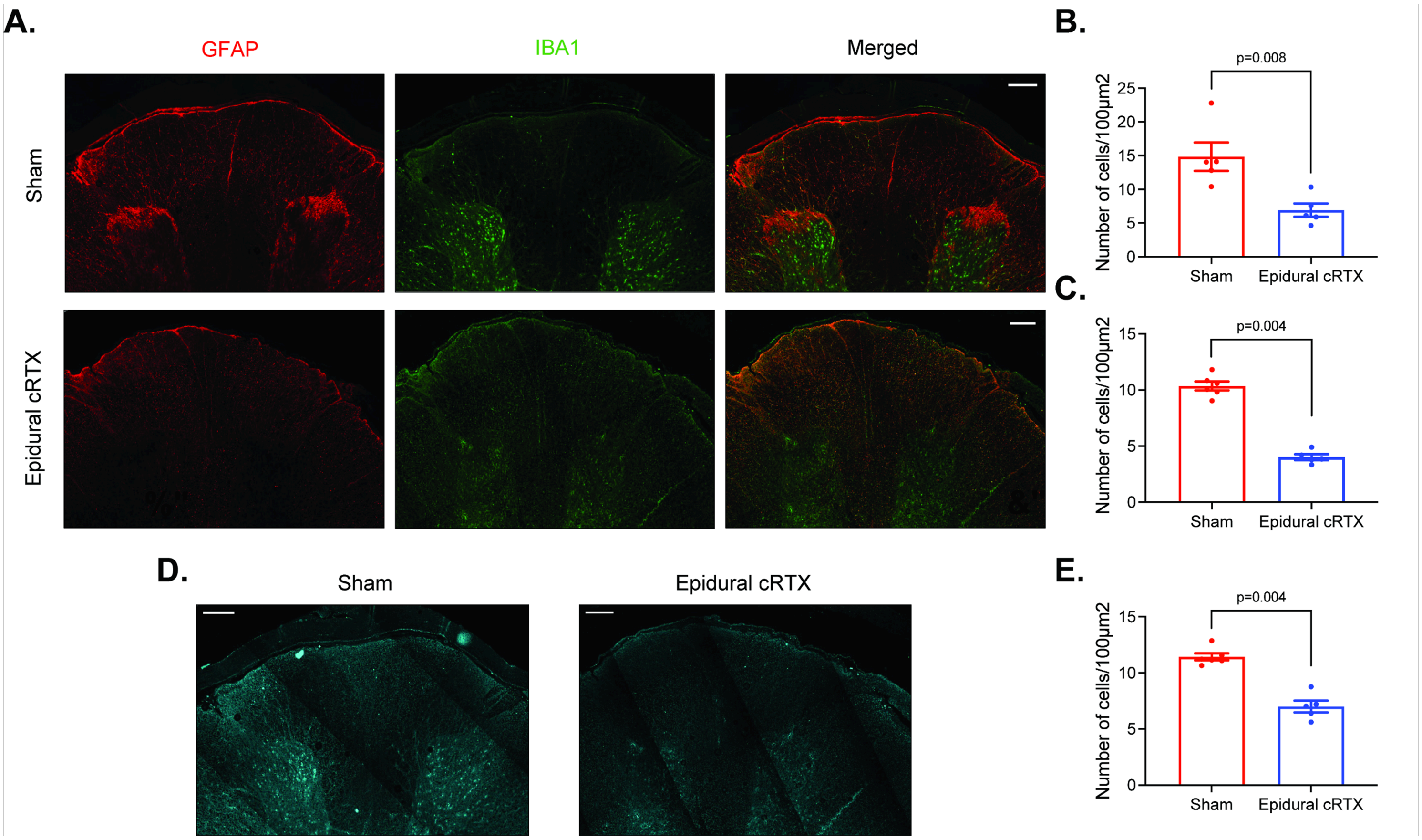

### Figure 6

**Immunohistochemical changes associated with ablation of cardiac spinal nociceptive nerves. (A)** Representative images of immunostaining of spinal horns in epidural cRTX and sham animals for GFAP and IBA1 (markers of glial activation and microglia, respectively). **(B-C)**Quantified data shows decreased immunoreactivity in epidural cRTX (*n=5*) *vs* sham (*n=5*) animals. **(D)**Representative images of immunostaining of the spinal horn for CD3 (marker of T cells). **(E)** Quantified data shows a significant reduction in the amount of CD3 positive T cells in epidural cRTX (*n=5*) *vs* sham (*n=5*) animals, suggesting reduced inflammation. Differences between sham and epidural cRTX animals were assessed using the Mann Whitney test. Scale bars represent 500 µm.

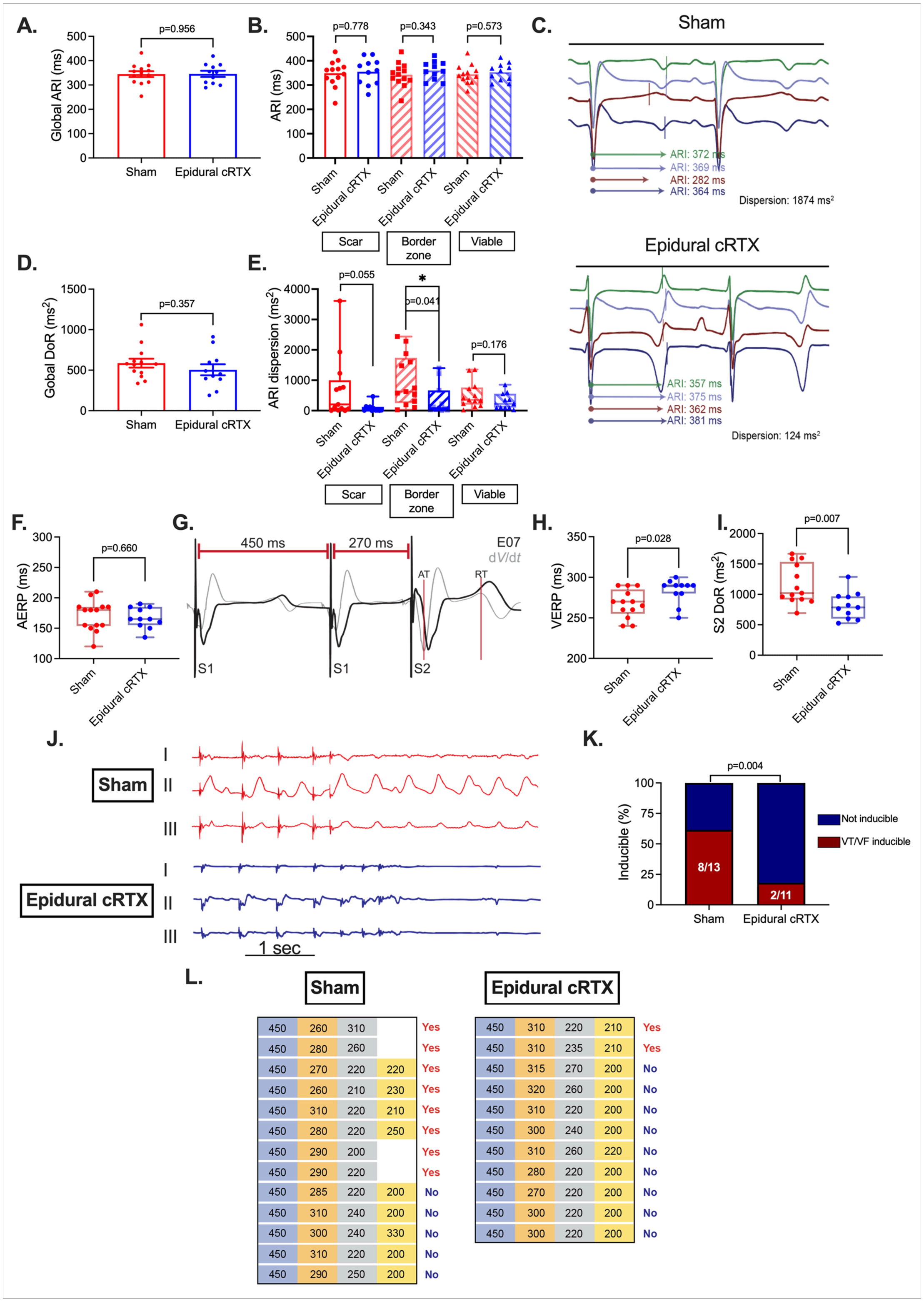

**Sham**

| | | | | |
|---|---|---|---|---|
| 450 | 260 | 310 | | Yes |
| 450 | 280 | 260 | | Yes |
| 450 | 270 | 220 | 220 | Yes |
| 450 | 260 | 210 | 230 | Yes |
| 450 | 310 | 220 | 210 | Yes |
| 450 | 280 | 220 | 250 | Yes |
| 450 | 290 | 200 | | Yes |
| 450 | 290 | 220 | | Yes |
| 450 | 285 | 220 | 200 | No |
| 450 | 310 | 240 | 200 | No |
| 450 | 300 | 240 | 330 | No |
| 450 | 310 | 220 | 200 | No |
| 450 | 290 | 250 | 200 | No |

**Epidural cRTX**

| | | | | |
|---|---|---|---|---|
| 450 | 310 | 220 | 210 | Yes |
| 450 | 310 | 235 | 210 | Yes |
| 450 | 315 | 270 | 200 | No |
| 450 | 320 | 260 | 200 | No |
| 450 | 310 | 220 | 200 | No |
| 450 | 300 | 240 | 200 | No |
| 450 | 310 | 260 | 220 | No |
| 450 | 280 | 220 | 200 | No |
| 450 | 270 | 220 | 200 | No |
| 450 | 300 | 220 | 200 | No |
| 450 | 300 | 220 | 200 | No |

### Figure 7

***Effects of sympathetic nociceptive afferent ablation on ventricular APD, DoR, refractoriness, and ventricular arrhythmia inducibility after chronic MI.*** **(A)** Administration of thoracic epidural RTX did not alter global baseline activation recovery intervals (ARI), nor **(B)** regional ARIs four to six weeks post-MI. **(C)** Examples of border zone electrograms from a sham (top) and epidural cRTX (bottom) animal showing ARIs from these regions. **(D)** In epidural cRTX animals, global dispersion of repolarization (DoR)

was not significantly different from sham animals, however, **(E)** regional analyses demonstrated that ARI dispersion was greatest in border zone regions in both sham and epidural cRTX animals, but that this border zone dispersion was significantly less in the epidural cRTX animals, compared to sham animals. **(F)** Atrial ERP (AERP) was not significantly different between Sham and epidural cRTX animals. **(G)**However, ventricular ERP (VERP) was significantly prolonged in epidural cRTX *vs* sham animals. **(H)** Example of ventricular ERP (VERP) measurements showing the last-captured extra-stimulus (S2) at interval of 270 msec. **(I)** RT-dispersion of the S2 paced beat was also significantly decreased in epidural cRTX animals compared to sham animals. **(J)** Example of VT/VF induction using programmed stimulation. The sham animal (upper panel) was inducible with 1 extra-stimulus, but the epidural cRTX animal (lower panel) was not inducible. **(K)** VT/VF inducibility was reduced from 62% in sham to 18% in epidural cRTX animals with up to four extra-stimuli. **(L)** Breakdown of stimulation parameters used for induction of VT/VF. All electrophysiological parameters were compared between sham and epidural cRTX animals using the unpaired Student's t-test, VT inducibility was compared by the exact binomial test. *n*=13 for sham and *n*=11 for epidural cRTX for all reported electrophysiological parameters.

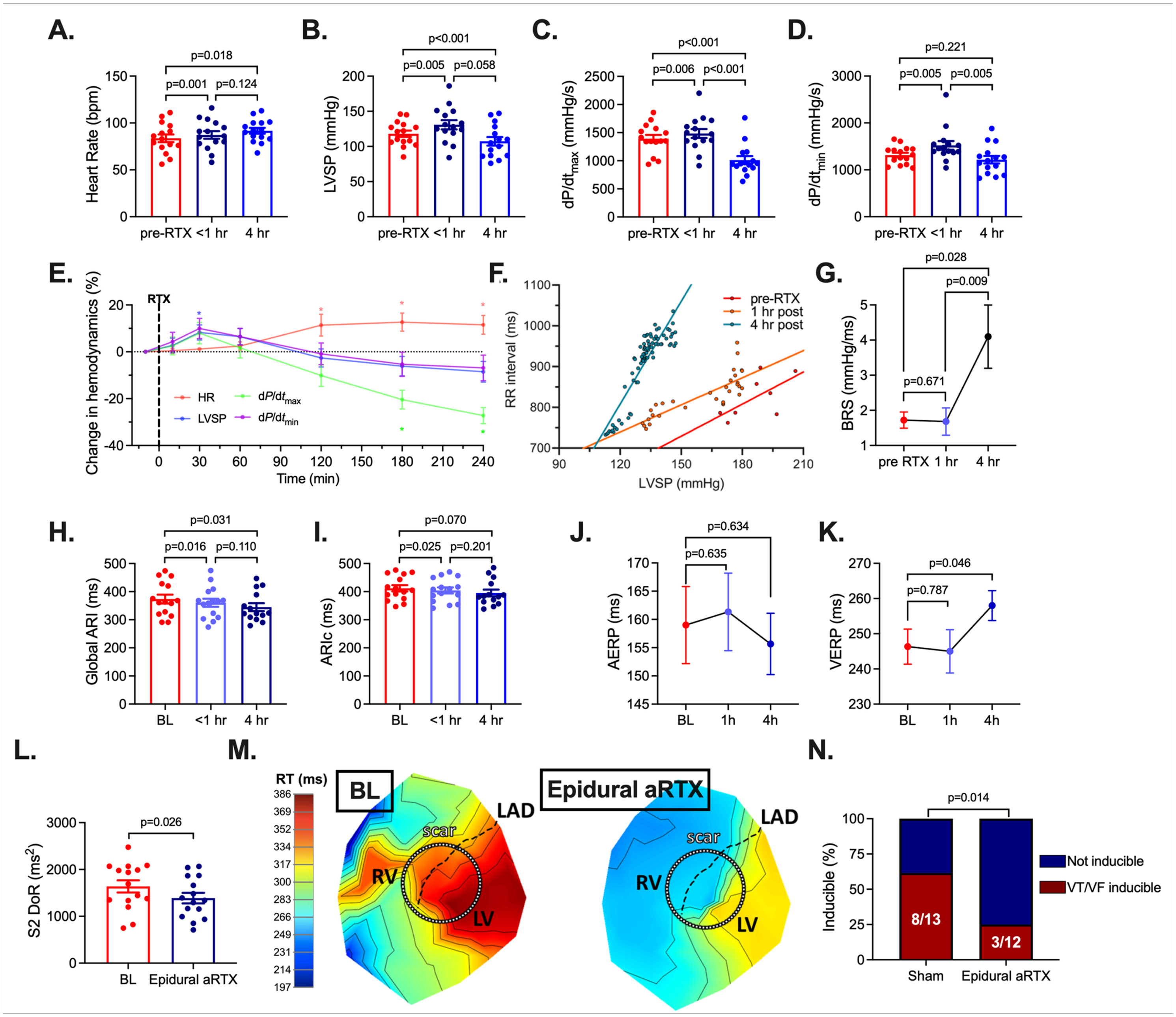

### Figure 8

***Temporal hemodynamic responses and electrophysiological effects of acute spinal nociceptive afferent ablation in the setting of chronic MI.*** The acute effects of epidural RTX were assessed to determine the kinetics and tolerability of RTX in infarcted animals (*n*=15). Within 1-hour of administration, RTX increased **(A)** HR, **(B)** left ventricular systolic pressure (LVSP), **(C)** left ventricular inotropy ($dP/dt_{max}$), and **(D)** LV lusitropy ($dP/dt_{min}$). LV lusitropy remained significantly elevated, whereas LV inotropy and LVSP were decreased 4 hours after epidural RTX administration. **(E)** Percentage change in HR, LVSP, LV inotropy and lusitropy over time are shown. **(F)** Beat-to-beat changes in RR interval *vs* LVSP were plotted to assess baroreflex sensitivity (BRS) before, 1-hour, and 4-hours post-RTX injection. **(G)** BRS was significantly improved by 4-hours post-RTX (*n*=10). **(H)** Acute epidural RTX shortened activation recovery intervals (ARI) at 1- and 4-hours after administration. **(I)** When corrected for heart rate (ARIc), this effect was no longer significant. **(J)** Epidural RTX did not alter atrial ERP (AERP) in chronic MI animals. **(K)** Epidural RTX prolonged VERP 4-hours after administration. **(L-M)** This was accompanied by a reduction in the S2 dispersion of repolarization time (DoR). **(N)** While 8/13 sham animals were inducible for sustained VT/VF, only 3/12 epidural aRTX animals were inducible at 4 hours post-RTX administration. Serial hemodynamic and electrophysiological parameters were compared by repeated-measures ANOVA. Serial BRS was compared using the Friedman test and RT dispersion was compared using a paired Student's t-test. Inducibility was compared using the exact binomial test. RV: Right ventricle, LV: Left ventricle, LAD: Left anterior descending artery.

## Supplementary Files

This is a list of supplementary files associated with this preprint. Click to download.

- SupplementaryDataFile1.xlsx
- SupplementaryDataFile2.xlsx
- 16032025SupplementalFigures.docx
